# Impact of electrode drying time on capacitive performance of honey-derived graphene nanosheets for supercapacitors

**DOI:** 10.1038/s41598-026-62039-8

**Published:** 2026-07-19

**Authors:** Ahmed Amer Khafaga, Ahmed A. El-Hamalawy, Mohammed Said Mohammed Abu-Elmagd, Sameh Hassan

**Affiliations:** 1 Engineering Mathematics and Physics Department, Higher Institute of Engineering, El-Shorouk Academy, El Shorouk, Egypt; 2 Physics Department, Faculty of Science, Menoufia University, Shebin El-Koom, 32511 Menoufia Egypt; 3 Physics Department, Faculty of Science, El Sadat City University, El Sadat City, 32897 Menoufia Egypt

**Keywords:** Honey-derived graphene, Hierarchical pore regulation, Electrochemical energy storage, Drying time optimization, Supercapacitor electrodes, Biomass-derived graphene, Chemistry, Energy science and technology, Materials science, Nanoscience and technology

## Abstract

Graphene nanosheets have a significant impact in the energy storage field, particularly in the realm of supercapacitors and capacitive deionization, due to their exceptional properties. This research aims to develop a facile method for preparing graphene nanosheets from biomass and examine the influence of the electrode drying time on the electrochemical properties of the prepared electrodes. Honey, as a carbon source, offers advantages over typical biomass because of its uniform composition, high carbon content, and ability for controlled low-temperature carbonization, which improves porosity and wettability. This chemical process was followed by KOH chemical activation with N_2_ gas injection. The physical and chemical properties of graphene nanosheets were examined with X-ray diffraction (XRD), X-ray photoelectron spectroscopy (XPS), scanning electron microscopy (SEM), energy-dispersive X-ray (EDX), Transmission Electron Microscope (TEM), low-temperature nitrogen adsorption-desorption for isothermal characterization, zeta potential, and particle size measurements. The honey-based graphene nanosheets have a specific surface area of 1427 m² g⁻¹ and a pore volume of 0.654 cm³g⁻¹. Consequently, the electrochemical performance of the prepared electrodes was assessed via galvanostatic charge-discharge (GCD), electrochemical impedance spectroscopy (EIS), and cyclic voltammetry (CV). However, the optimum drying time (24 h) had a significant effect on the specific capacitance of electrodes (maximum of 240 Fg⁻¹), which was achieved at a current density of 0.3 Ag⁻¹ when tested in a 0.5 M Na_2_SO_4_ aqueous electrolyte. These results present promising rate capability and competitive performance for supercapacitor applications compared with other graphene-based supercapacitor electrodes. Moreover, this sustainable and cost-effective method may enhance the performance of supercapacitor electrodes.

## Introduction

Energy storage is one of the most important targets that researchers focus on to meet global needs. Supercapacitors are highly distinctive energy storage devices owing to their inherent operational benefits, which include enhanced thermal safety, long-term cyclic endurance, high power density, rapid charge-discharge kinetics, and near-perfect coulombic reversibility. Thereby, supercapacitors show an alternative candidate for next-generation energy storage. Moreover, supercapacitors are particularly well-suited for demanding applications requiring immediate, high-current delivery, like electric vehicles, owing to their non-explosive nature and ability to provide substantial power bursts^1,2^. The charging process is significantly faster than traditional batteries and can withstand millions of charge-discharge cycles, far exceeding the lifespan of conventional capacitors and batteries^3^.

Supercapacitance, a promising energy storage technology, operates through two primary mechanisms, Electric Double-Layer Capacitance (EDLC) and Pseudocapacitance, alongside hybrid systems that synergize both. EDLC, a non-faradaic process, leverages electrostatic charge separation at the electrode-electrolyte interface (forming a Helmholtz double layer) and excels in high-power applications due to rapid ion adsorption/desorption kinetics, with carbon-based materials like graphene offering ideal substrates. Pseudocapacitance, in contrast, relies on faradaic redox reactions at or near the electrode surface, as seen in transition metal oxides (e.g., RuO₂) or conductive polymers (e.g., PEDOT), delivering high energy density but often at the cost of reduced power density and cycle stability. Hybrid systems bridge these mechanisms by integrating EDLC’s fast kinetics with pseudocapacitance’s redox activity, exemplified by carbon-metal oxide composites^4–6^.

Consequently, Fabrication of electrode materials has an essential concern, particularly with desirable features such as strong chemical stability, optimum wet-ability, Suitable pore size distribution, larger specific surface area, and good electrical conductivity^4,7,8^, mainly focusing on finding different and inexpensive sources for those active materials^9^. Therefore, carbon-activated materials were presented with the potential to enhance supercapacitor electrodes. However, activated carbons can be extracted from biomass and organic residues to develop sustainable and cost-effective electrode materials^10^. such as hemp fiber^11,12^, rice husk^13,14^, corn-cob^15,16^, peat moss^17^, bagasse^18^, waste coffee grounds^19^, foxtail grasses^20^, willow catkin^21,22^, Enteromorpha^23^, loofah sponges^24^, olive pits^25^, chestnut shell^26^, pinecones^27^, peanut shell^28^, shiitake mushrooms^29^, red cedar wood^30^, tobacco waste^31^, soybean waste^32^, animal bone^33^, moringa fruit shells^34^, infested ash-trees^35^, melons^36^, starch^37^, fish scale^38^, moringa stems^39^. However, all of these materials present unique challenges in terms of synthesis and performance optimization.

Honey, a carbon-rich structure, has been a promising biomass to produce activated carbon materials for the last 10 years. Yang et al. created carbon dots (CDs) based on honey using a simple and environmentally friendly process for sensing and imaging applications^40^. On the other hand, Li, et al. produced activated carbon for supercapacitors by treating honey with varied KOH ratios and carbonizing it at 800 $\:^\circ\:C$ in a nitrogen atmosphere^41^. By 2017, Kiruthiga, et al. used different ratios of KOH in the argon atmosphere to produce activated carbon based on honey^42^. However, its application in graphene nanosheet synthesis remains mainly unexplored.

Graphene nanosheets can be used in energy storage applications, particularly in supercapacitors. However, using traditional biomass as a precursor often poses challenges, such as non-uniformity and difficulties in controlling the processing. Therefore, this work aims to present a unique and eco-friendly solution to produce graphene nanosheets from honey.

Honey, a widely available natural product, represents an innovative and significantly underexplored carbon source with remarkable potential for advanced nanomaterials. Unlike traditional biomass sources such as agricultural waste, wood, or marine materials, honey offers unique advantages for electrode material synthesis due to its homogeneous molecular composition and high carbon content efficiency. The high sugar content in honey, primarily fructose (38.2%) and glucose (31.3%), facilitates a unique carbonization process that naturally promotes the formation of sp2-hybridized carbon networks^41,42^. These characteristics enable more controlled carbonization at lower temperatures (800 °C vs. 900–1000 °C typically required for other bio-sources), resulting in a well-developed hierarchical porosity with balanced micro/mesopore distribution compared to wood-derived carbons^30^. This hierarchical structure combines micropores for high capacitance and mesopores for efficient ion transport, while the presence of oxygen-containing functional groups inherited from honey’s natural composition enhances the material’s wettability and electrode-electrolyte interactions. This structural advantage, combined with honey’s natural preservation properties, leads to enhanced electrochemical performance, demonstrated by competitive specific capacitance (200–240 F g^− 1^) compared to 120–190 F g^− 1^ for materials derived from sources like rice husk^13,14^, corncob^15,16^, and willow catkin^21,22^, and better cycling stability.

While honey has been explored as a precursor for activated carbon, its use in the straightforward creation of graphene nanosheets has not been extensively studied, especially concerning how electrode drying time impacts their capacitive performance. This research directly tackles this gap. We’re optimizing the electrode preparation by carefully controlling the drying process, which reveals new insights into how this seemingly simple step significantly affects the electrode’s structural integrity, ion transport, and overall electrochemical efficiency.

In this work, the natural biomass, honey, was used to prepare the graphene nanosheets using an enhanced method. The choice of honey is based on its high carbon content, reduced environmental impact, lower production costs (compared to the cost of treatment processes of other biomasses), and simplified processing. Also, their exceptional performance makes them a strong contender for outstanding performance in energy storage applications. Moreover, the intrinsic purity of honey minimizes the presence of unwanted impurities and reduces the need for extensive purification steps, leading to a more environmentally friendly synthesis process compared to conventional graphene production methods that often require harsh chemical treatments and multiple purification stages.

The enhanced implemented method succeeded in producing the Honey-Derived Graphene-Nanosheets (HD-GNS) by the carbonization of honey. After that, it is followed by the activation of the produced carbon powder, which results in a fragile carbon structure. Then, it was subjected to continuous grinding and purification processes before preparing electrodes and characterization processes. This output material has been targeted for preparing supercapacitor electrodes. Consequently, the influence of electrode-drying time on electrochemical characteristics.

## Materials and methods

### Materials

Honey was used as a biomass. The chemical materials were granules of potassium hydroxide $\:\left(KOH\right)$, $\:1M\:$Hydrochloric acid$\:\:\:\left(HCl\right)$, stainless steel sheet, nitrogen gas (N_2_), concentrated sulfuric acid $\:{(H}_{2}{SO}_{4})$, N-methyl-2-pyrrolidone (NMP) (LOBA Chemie) as a solvent, Polyvinylidene fluoride (PVDF) of molecular weight ~ 275,000 (pellet shape from Sigma-Aldrich, USA), and sodium sulfate $\:\left({Na}_{2}{SO}_{4}\right)$. Double-distilled water (DDW) was collected using a glass distiller (Fi-streem water still) and used for all solution preparation and washing elements.

### Experimental procedures

#### Synthesis of honey-derived graphene nanosheets (HD-GNS)

**Fig. 1:**
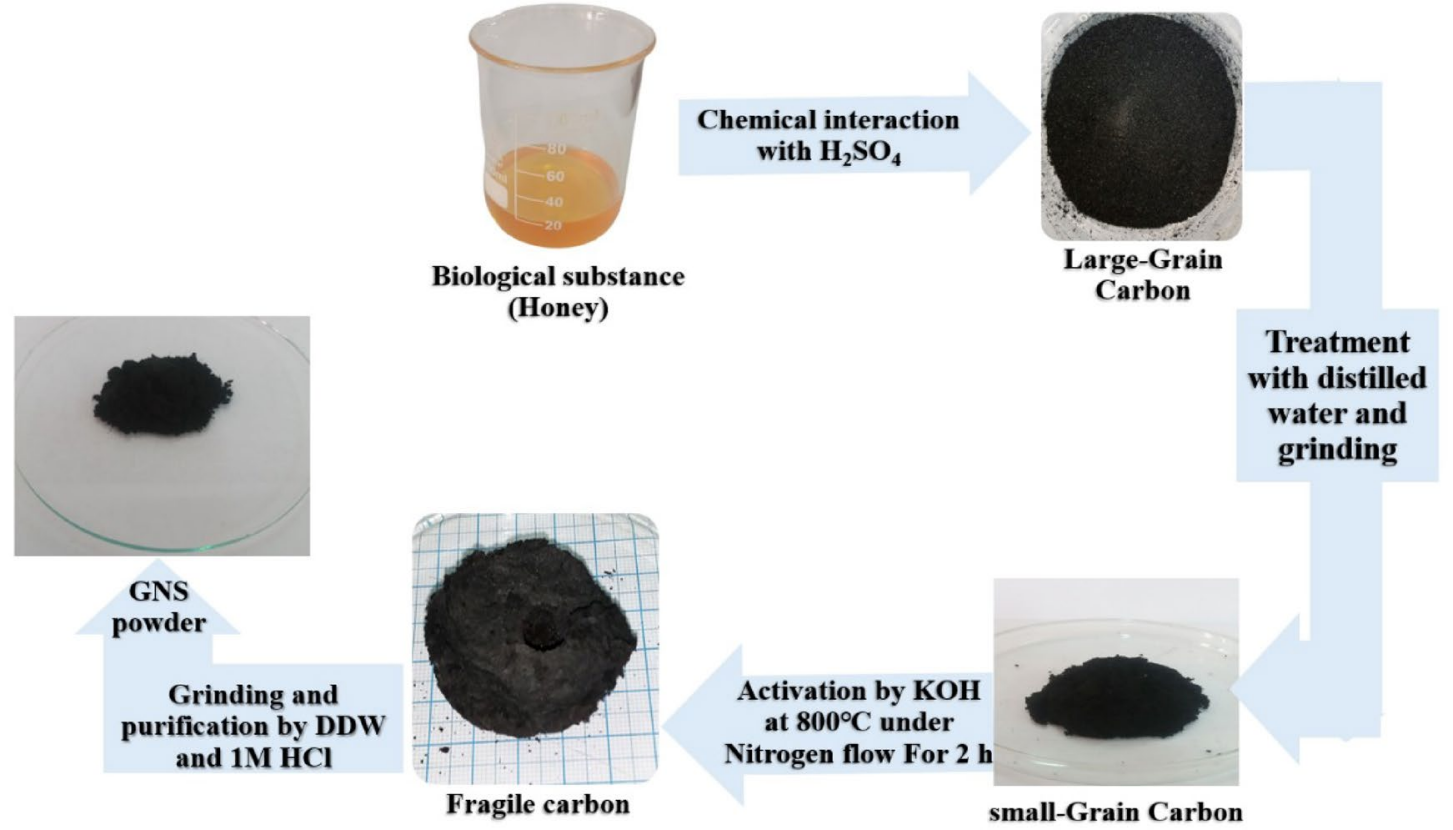
Schematic diagram for synthesizing GNS material.

The process of graphene nanosheet preparation is shown in Fig. 1 concentrated $\:{H}_{2}{SO}_{4}$ was gradually added to pure honey in a (50 ml) glass beaker under continuous stirring until the reaction was initiated, continuing for 10 min to extract the carbon. To remove reaction residues, the resulting carbon was washed multiple times with double-distilled water, followed by drying at 80 °C for 12 h.

The dried carbon was then subjected to continuous grinding to achieve a fine powder before being mixed with ground potassium hydroxide (25% carbon: 75% [missing chemical name, e.g., KOH]) using continuous milling in an agate mortar. Subsequently, the mixture was heated in a furnace at 800 °C for 2 h at a heating rate of 5 °C/min under a continuous flow of Nitrogen (N_2_) gas.

After that, the sample was left to reach 300 °C inside the furnace with continuous gas flow and then to room temperature without gas injection. As a result, a black fragile carbon structure was formed, which was subjected to continuous grinding and washing with deionized water, which was followed by an acidic modification process with controlled pH (~ 7 by using a digital pH meter). Finally, this modification is followed by a filtering and drying process at 100℃ for 24 h and milling again to ensure that all granules have the same particle size.

#### Characterization of HD-GNS

X-ray Diffraction (XRD) analysis was conducted using a Panalytical Empyrean 3 instrument “Model name Malvern Panalytical Empyrean 3 (3rd Generation)” (Malvern, Netherlands) equipped with Cu Kα radiation (λ = 0.15406 nm) operating at 40 kV and 0.030 A. Scans were performed across a 2θ range from 5° to 80°. The primary objective of this XRD examination is to analyse a material’s crystallographic structure, specifically identifying crystalline phases, determining lattice parameters, assessing crystallinity, and evaluating grain or crystallite size.

X-ray Photoelectron Spectroscopy (XPS), performed using a Thermo-Fisher Scientific (USA) instrument “Model name Thermo Scientific K-Alpha XPS spectrometer”, was utilized to investigate the materials’ atomic composition and chemical functional groups. Conducted with a monochromatic Al K-alpha X-ray source under 10⁻⁹ millibar pressure, and parameters including a -10 to 1350 eV energy range, 400-micrometer spot size, 200 eV full spectrum pass energy, and 50 eV narrow spectrum pass energy, the primary objective of XPS was to characterize the graphene nanosheet surface chemistry by detailing its elemental composition, identifying specific carbon and oxygen bonding environments, and detecting any minor impurities, thereby providing a comprehensive assessment of the surface’s chemical states and purity.

The textural properties of the synthesized HD-GNS were thoroughly investigated using total isothermal characterization techniques. Brunauer-Emmett-Teller (BET) analysis was employed to determine the material’s surface area, while the pore structure was elucidated through N₂ adsorption/desorption measurements conducted at 77.35 K. Before these surface measurements, a 0.0209 g sample of the prepared powder was degassed under vacuum at 300 °C, with a heating rate of 20 °C/min for 120 min. Additionally, the pore size distribution was evaluated using both density functional theory (DFT) and the Horvath-Kawazoe (HK) methods. All these analyses were performed using an Anton Paar NOVA 800 instrument “Model, St 1 on NOVA 800 [s/n:1050044426]” located in Egypt.

To evaluate the microstructural features of the synthesized HD-GNS, transmission electron microscopy (TEM) was performed using a JEOL JEM-2100 Plus system at an accelerating potential of 200 kV. The sample preparation involved ultrasonically dispersing the HD-GNS powder in ethanol and casting the suspension onto a conventional carbon-coated copper grid.

Zeta potential and particle size measurements (Zeta-sizer Nano ZS, Malvern, UK) are essential for characterizing a material’s colloidal stability and surface attributes. Zeta potential primarily assesses particle surface charge and potential, revealing insights into electrostatic interactions and repulsive behaviour in ionic suspensions; a higher value generally denotes greater slurry stability and less aggregation. Concurrently, particle size measurements determine the physical dimensions of grains, complementing zeta potential data to clarify factors affecting overall dispersion and aggregation quality.

#### Electrode preparation

Here, the produced GNS slurry was prepared with a percentage of 90% (9 mg) and polyvinylidene fluoride (PVDF) 10% (1 mg) as binder material dissolved in 15 µL N-Methyl-2-pyrrolidone (NMP) without adding any conductive additive, followed by sonication for 30 min at a temperature of 60 ℃. The slurry was spread on a 1 cm^2^ area of a stainless-steel sheet with dimensions 2 cm x 1 cm x 0.3 mm with 3.2 mg cm^− 2^ mass loading. The prepared samples were dried at 100 ℃ for 2 and 24 h. The selection of these particular drying periods aimed to explore how initial, rapid solvent removal (2 hours) compares to a more extended and thorough solvent evaporation (24 h) in influencing the electrode’s structural integrity, porosity, and electrochemical behaviour. The primary objective was to ascertain the effectiveness of residual solvent removal and its subsequent implications for ion accessibility and charge transfer within the electrode material.

#### Electrochemical characterization of the electrode

The prepared electrodes were examined electrochemically using different analysis techniques. Firstly, galvanostatic charge and discharge (GCD) experiments were recorded at different actual currents, ranging from 1 to 10 mA, using a voltage window of 0 to 1 V. The second technique is cyclic voltammetry (CV), which is performed with different scan speeds ranging from 10 to 100 mV per second at a voltage range of (0–1) V. Finally, the electrochemical impedance spectroscopy (EIS) was measured in the frequency range of 10 mHz to 100 kHz, with 10 mV alternating current amplitude, superimposed on 10 mV DC voltage. These three techniques were performed in a 0.5 M Na_2_SO_4_ electrolyte using a three-electrode system by instrument (Biologic workstation SP-150 potentiostat/galvanostat). The prepared active electrode material was set as a working electrode. A platinum rod works as a counter electrode, and a saturated Ag/AgCl (KCl saturated) is used as a reference electrode. All data for these procedures were acquired using the compatible SP-150 software (EC-Lab version 11.43).

The specific capacitance (C_sp_) in F/g can be determined using each technique, as in Eq. (1), from the charge-discharge current (I) divided by the rate of voltage change $\:\frac{\varDelta\:V}{\varDelta\:t}$, and active material mass (m) of the tested electrode.

1 $$\:{C}_{sp}\left(GCD\right)=\frac{I}{m(\varDelta\:V/\varDelta\:t)}$$

Also, (C_sp_) is calculated using the cyclic voltammetry data as in Eq. (2), using the capacitive charge (Q), derived by dividing the entire area of the CV curve by two, divided by the product of mass of the active electrode and width of the potential window (V) as,

2 $$\:{C}_{sp}\left(CV\right)=\frac{Q}{mV}$$

Where:

3 $$\:Q=\frac{{Q}_{anodic}+{Q}_{cathodic}}{2}$$

$\:{\mathrm{Q}}_{\mathrm{a}\mathrm{n}\mathrm{o}\mathrm{d}\mathrm{i}\mathrm{c}\:}$and $\:{\mathrm{Q}}_{\mathrm{c}\mathrm{a}\mathrm{t}\mathrm{h}\mathrm{o}\mathrm{d}\mathrm{i}\mathrm{c}}\:$denotes the anodic and cathodic voltammetric charge. Also, from the impedance technique, (C_sp_) is evaluated from the (EIS) curve according to Eq. 4 using the imaginary impedance Z_im_ and the lowest frequency (f_min_).

4 $$\:{C}_{sp}\left(EIS\right)=\frac{1}{mx2\pi\:{f}_{min}x\left|{Z}_{im}\right|}$$

To maintain strict analytical consistency, all gravimetric specific capacitance C_sp_, specific energy density (E), and specific power density (P) metrics presented in this study are computed relative to the net active material mass (m) fixed onto the single working electrode. For evaluations conducted within the three-electrode cell matrix, this mass metric corresponds specifically to the electrochemically active carbon matrix cast onto the individual working substrate. Conversely, for the symmetric two-electrode assembly, performance profiles are rigorously standardized against the total active mass of both opposing electrode elements (M = m_anode_ + m_cathode_ = 2 m), ensuring that the reported parameters reflect standardized full-cell benchmarking boundaries rather than full packaged device mass.

#### Electrochemical stability of the electrode

Assessing an electrode’s electrochemical stability is crucial for ensuring its long-term performance and reliability within an electrochemical system. This analysis primarily aims to confirm the electrode’s ability to maintain its structural and functional integrity throughout extended operation within an electrochemical system, defining its operational voltage window and preventing undesirable side reactions such as decomposition or corrosion. This ongoing stability ensures consistent and efficient charge storage over time, which is vital for practical applications.

## Results and discussion

### Characterization of honey-derived graphene nanosheets (HD-GNS)

#### X-ray diffraction (XRD) analysis

**Fig. 2:**
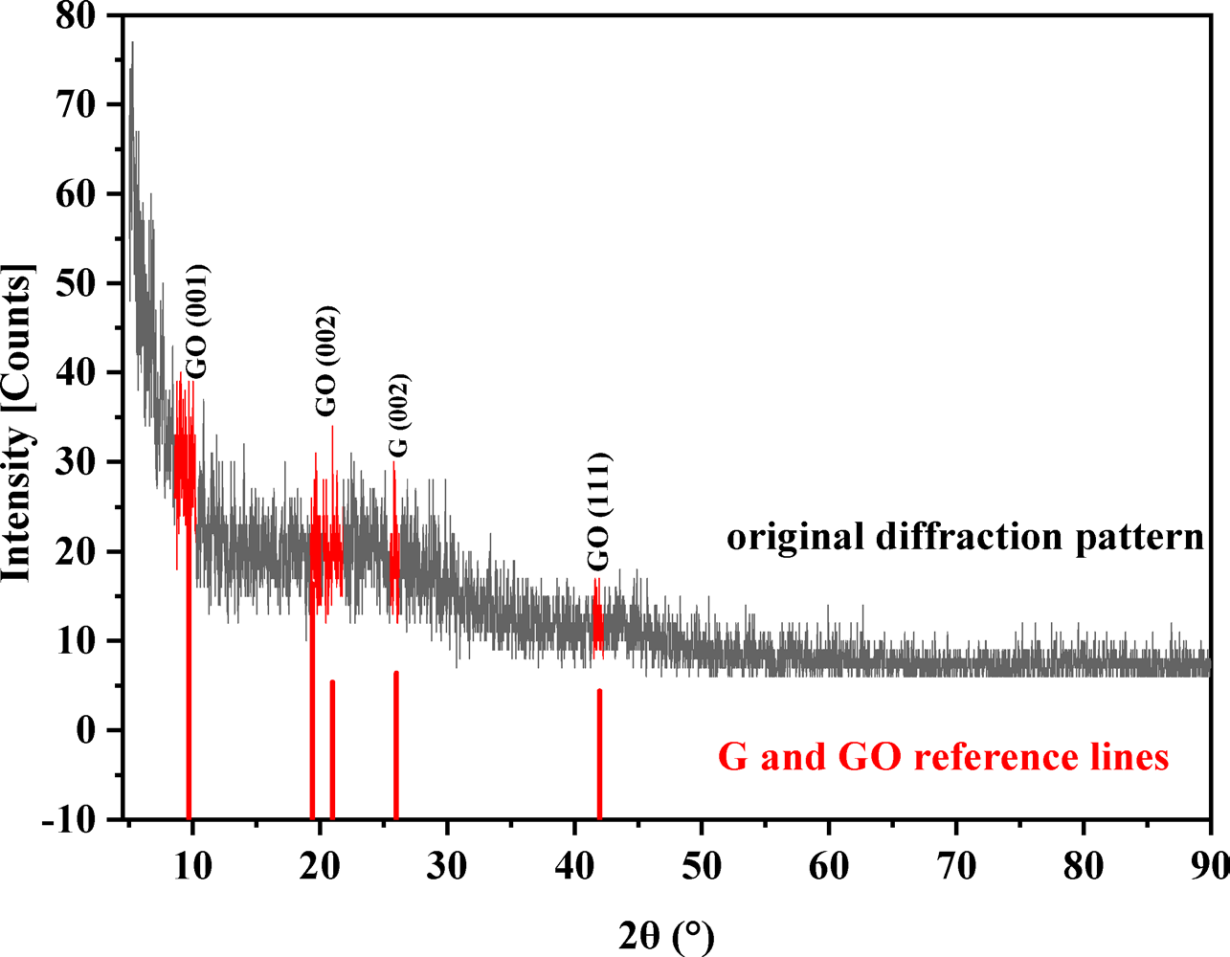
XRD pattern for the HD-GNS sample with graphene and graphene oxide baseline.

The structural framework and crystallinity of the synthesized graphene nanosheets (GNS) were investigated using XRD analysis, as illustrated in Fig. 2. The diffractogram displays a diffuse, broad reflection centered between 2Ɵ = 22° − 25°, which corresponds to the (002) crystallographic plane. This prominent line broadening confirms a highly disordered, turbostratic carbon architecture lacking long-range crystalline periodicity, a stark contrast to the sharp, intense peak typically observed at 26.5 degrees 2-theta for perfectly aligned, parallel layers in pristine graphite. This structural disruption and minor peak shift highlight a significant degree of layer exfoliation within the honey-derived graphene nanosheets, implying that individual graphene sheets are not densely stacked. The XPS results strongly validate this widespread structural disorder. From an electrochemical standpoint, this loose, turbostratic configuration is particularly advantageous for supercapacitor applications; the random layer orientation effectively suppresses tight face-to-face sheet restacking, thereby preserving the material’s massive specific surface area. This open configuration increases the accessible interlayer spacing, facilitating unhindered ion mobility and maximizing electrostatic double-layer charge accumulation.

Further evidence of a disordered carbon framework with restricted in-plane crystalline order comes from a faint and broad peak at approximately 43–44 degrees 2-theta, corresponding to the (100) or (101) planes. These peaks would also exhibit greater sharpness in highly crystalline graphite. The overall diffuse nature of the diffraction pattern implies that the material likely comprises a small number of graphene layers or highly imperfect individual sheets with numerous structural inconsistencies. Paradoxically, such irregularities can enhance the material’s active site density and specific surface area factors critical for achieving high capacitance in supercapacitors. The absence of sharp, well-defined graphite peaks confirms the effective transformation of the honey precursor into a graphene-like substance, circumventing the formation of large, crystalline graphite structures during fabrication. This outcome validates the efficacy of the chosen chemical carbonization and activation techniques in yielding a material with structural attributes ideally suited for energy storage.

The X-ray Diffraction (XRD) pattern exhibits specific diffraction peaks characteristic of graphene oxide (GO). Reflections are prominently observed at distinct 2-theta angles, corresponding to the GO (001), GO (002), and GO (111) crystallographic planes^43^, as depicted in Fig. 2. These peaks serve as definitive evidence for the presence of graphene oxide^44,45^, which forms as a result of oxygenated functional groups generated during the chemical oxidation of the graphite precursor. Despite the subsequent high-temperature activation at 800 °C, the persistence of these GO peaks indicates that complete reduction to pure graphene did not occur. Consequently, the synthesized material retains some characteristics of graphene oxide, attributed to the initial chemical oxidation process involving sulfuric acid. The relatively subdued sharpness of these GO peaks further indicates a turbostratic disorder, characterized by random rotations between adjacent graphene layers. Calculations using Bragg’s Law, (Eq. 5) (with *n* = 1) determined the interlayer spacing to be 0.911 nm for the (001) plane and 0.457 nm for the (002) plane.

5 $$\:d=\:\frac{n\lambda\:}{2*\:sin\:\theta\:}$$

Furthermore, the XRD pattern also displays a distinct graphene peak at 26.47 degrees 2-theta, corresponding to the (002) plane of graphene^43–46^. The GO nanosheet peaks appear notably broader and weaker, suggesting a random orientation of the graphene sheets in multiple directions—a characteristic consistent with the corrugated graphene nanosheet structure observed through SEM imaging. The presence of the 26.47° peak specifically confirms the successful reduction of some graphene oxide, indicating the formation of graphene sheets within the prepared sample^43,46^.

#### X-ray photoelectron spectroscopy (XPS) analysis

**Fig. 3:**
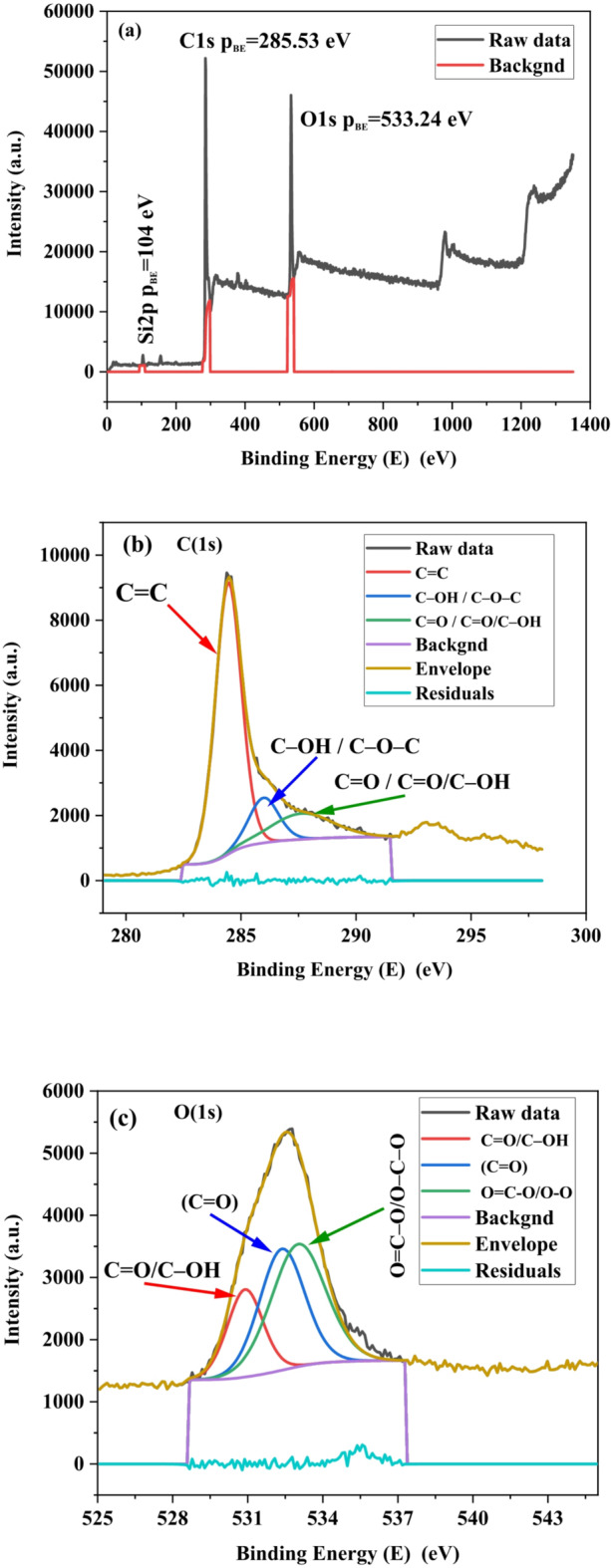
X-ray photon electron spectroscopy (XPS), (**a**) XPS survey wide scan spectra of HD-GNS sample, (**b,c**) deconvolution of C1s, and O1s peak in XPS survey spectra of HD-GNS.

As summarized in Table 1 and depicted in Fig. 3, the XPS survey spectrum consistently shows carbon as the primary element (e.g., 76.67 atomic %), complemented by a notable oxygen content (e.g., 20.62 atomic %). Minor impurities, such as silicon (e.g., 2.71 atomic % from silicon oxide), were also identified, providing a comprehensive assessment of surface purity.

Crucially, the XPS analysis not only confirms the high degree of graphitization, verifying the successful formation of the graphene structure, but also highlights the presence of various oxygen-containing functional groups on the HD-GNS. These functional groups are expected to significantly enhance the wettability of the graphene nanosheet surface in a water-based electrolyte. This improved wettability leads to a larger effective surface area, which is vital for efficient charge storage and strengthens the interactions between the electrode and the electrolyte. The specific arrangement and distribution of these oxygen functionalities directly contribute to the material’s overall electrochemical performance by offering additional sites for charge accumulation and facilitating smooth ion transport.

### Carbon bonding (C1s spectrum)

High-resolution deconvolution of the C1s spectrum is crucial for understanding the material’s graphitization and the nature of its carbon-oxygen bonds. A dominant peak at 284.48 eV, contributing 70.61% of the C1s signal (Fig. 3b), is characteristic of sp2-hybridized carbon atoms in graphitic C = C bonds. This high proportion directly indicates a substantial degree of graphitization within the nanosheets, which is essential for ensuring high electrical conductivity vital for high-performance supercapacitor electrodes. Additional C1s components at higher binding energies indicate carbon atoms bonded to oxygen: a peak at 285.98 eV (13.39%) corresponds to C-OH/C-O-C (hydroxyl/epoxy groups), while another at 287.63 eV (16%) is attributed to C = O/O-C = O (carbonyl/carboxyl groups)^47–53^. These oxygenated functionalities are critical for influencing surface properties.

### Oxygen bonding (O1s spectrum)

The O1s spectrum complements the C1s data by detailing the various oxygen-containing functional groups. Deconvolution of the O1s peak typically reveals three main components: peaks at 530.9 eV (20.45%), 532.37 eV (35.91%), and 533.02 eV (43.63%) (Fig. 3c). These correspond to C = O/C-OH, carbonyl (C = O) groups, and O = C-O/O-C-O functional groups, respectively^53–56^. A peak at slightly higher binding energies (e.g., 533.0-534.0 eV) might indicate adsorbed water, as suggested by the presence of silicon, Si-O bonds from silicon oxide impurities.

The XPS results collectively confirm a high level of graphitization, as indicated by the dominant C = C peak. This robust graphitic structure provides the necessary electrical conductivity for efficient charge transport. Simultaneously, the significant presence and diverse nature of oxygen-containing functional groups (C-OH, C-O-C, C = O, O-C = O) are paramount. These polar functionalities increase the surface energy of the graphene nanosheets, thereby substantially enhancing their wettability in aqueous electrolytes. Improved wettability ensures effective electrolyte penetration into the electrode’s porous structure, maximizing the contact area between the active material and electrolyte ions. This optimized interaction is fundamental for efficient ion propagation and transportation, leading to an expanded electrochemically active surface area for charge storage. In short, balancing high electrical conductivity via graphitization with improved surface wetting through functionalization determines the strong specific capacitance and efficient rate handling of the honey-sourced graphene electrodes.

**Table 1:** Chemical composition of HD-GNS from the XPS analysis.

Scan	Function group		Peak BE (eV)	FWHM (eV)	Area (*P*) CPS.eV	Atomic %
C1s	C = C		284.45	1.33	12002.55	70.61
	C–OH / C–O–C		285.98	1.51	2274.33	13.39
	C = O / C = O/C–OH		287.63	3.17	2715.77	16
O1s	C = O/C–OH		530.9	1.68	2567.02	20.45
	(C = O)		532.37	2.13	4503.59	35.91
	O = C–O/O–C–O		533.02	2.56	5469.56	43.63

#### Morphological property

**Fig. 4:**
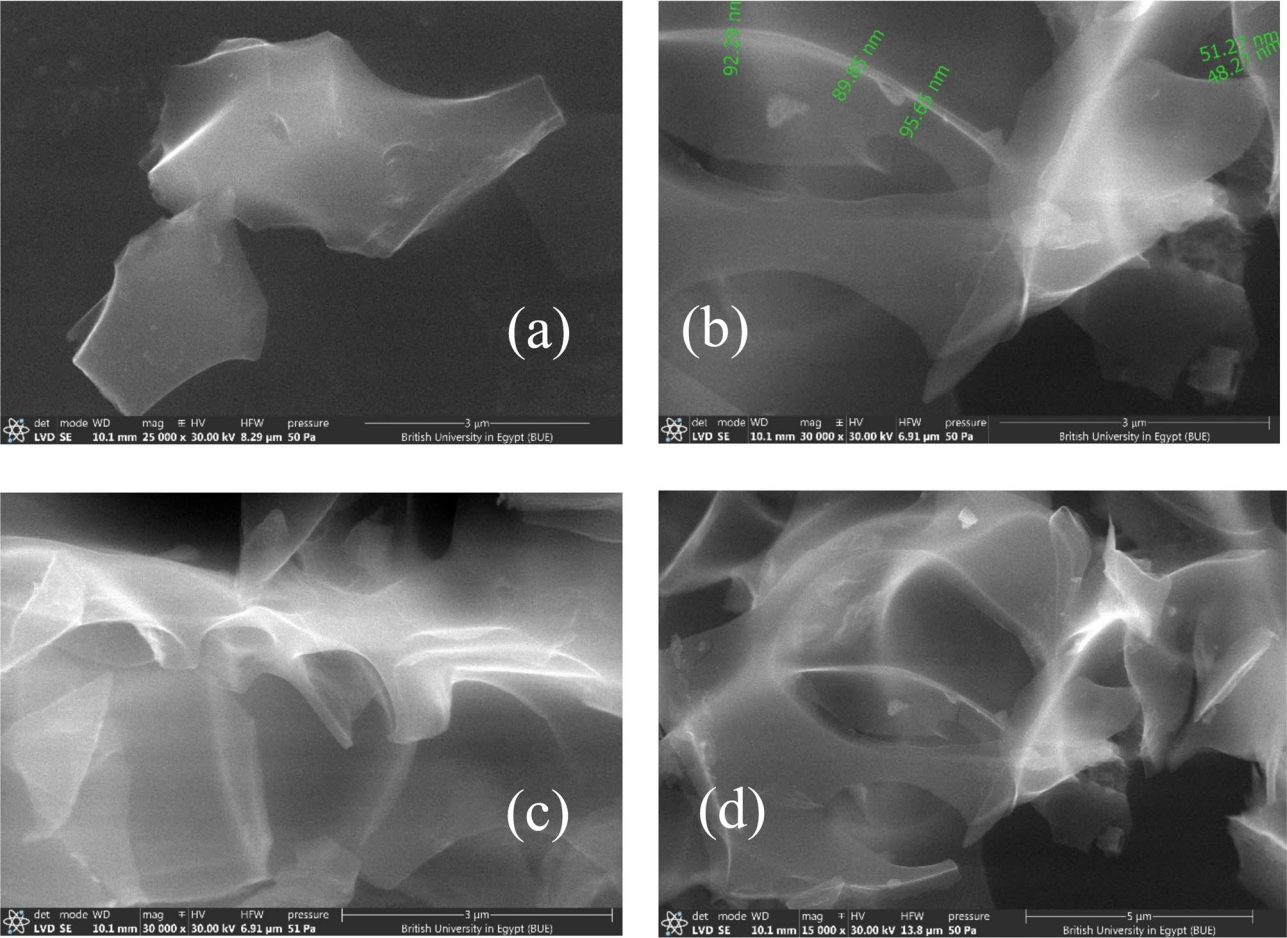
(**a-d**) SEM images for HD-GNS layers powder.

The morphology of the HD-GNS was thoroughly examined using Scanning Electron Microscopy (SEM) and Energy Dispersive X-ray (EDX) analysis. As shown in the SEM images (Fig. 4a-d), scans from various locations within the powder sample revealed a distinctive layered structure. This structure comprises multiple thin, combined, and crumpled sheets that collectively form a three-dimensional, semi-repeated network. These nanosheets exhibited varying thicknesses, typically ranging between 48.2 nm and 95 nm.

**Fig. 5:**
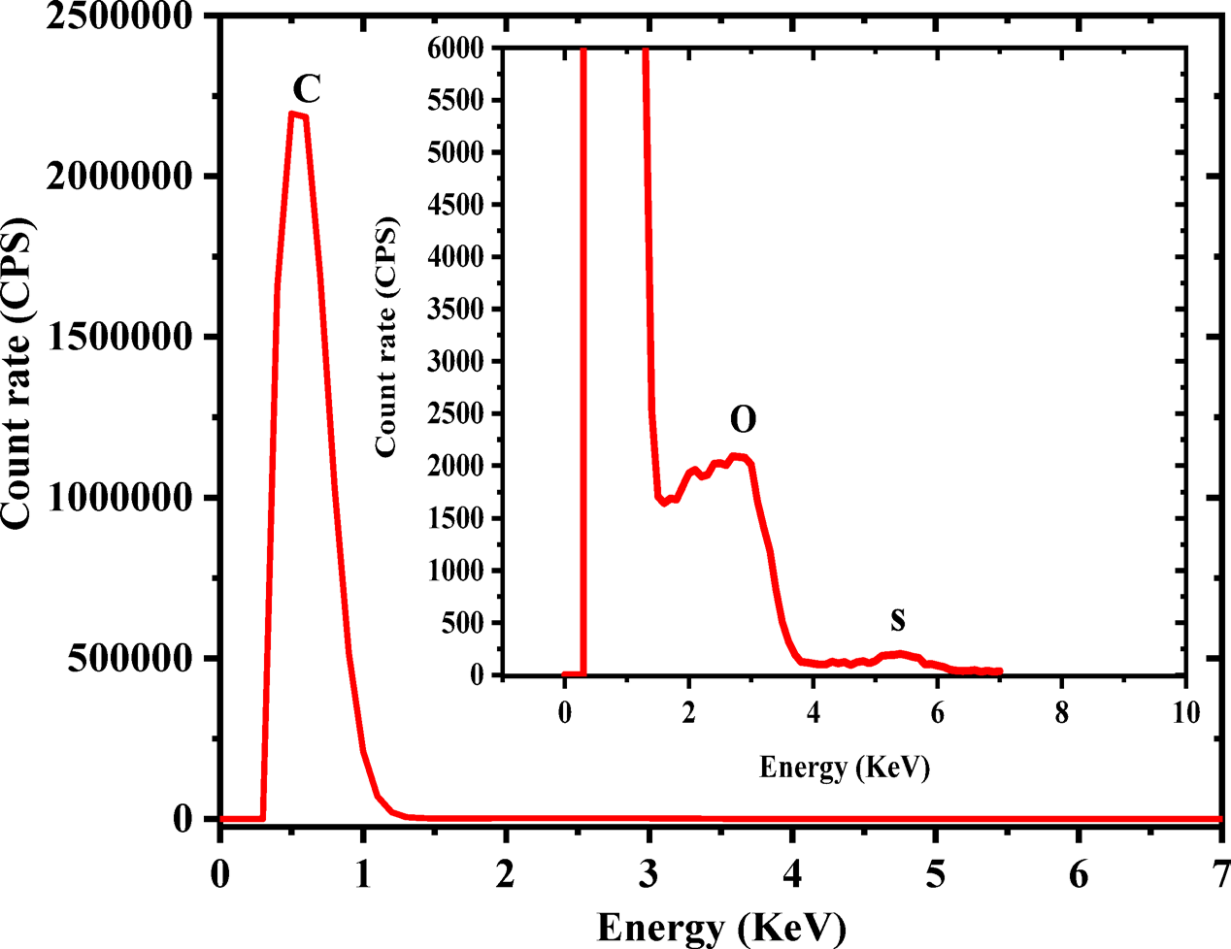
EDX spectroscopy for GNS layers.

Furthermore, EDX spectra (Fig. 5) identified the primary elemental composition, with carbon (C) and oxygen (O) being the most abundant elements, present at 75.41 weight% and 24.39 weight%, respectively. A small quantity of sulfur (0.2%) was also detected among other trace elements, as summarized in Table 2. These findings collectively confirm the successful formation of a nanolayered graphene sheet structure.

**Table 2:** Prepared GNS sample contaminated components.

Element	Weight%	Atomic %	Error %
*C*	75.41	80.4	4.73
*O*	24.39	19.52	13.63
*S*	0.2	0.08	33.44

#### Transmission electron microscope (TEM)

**Fig. 6:**
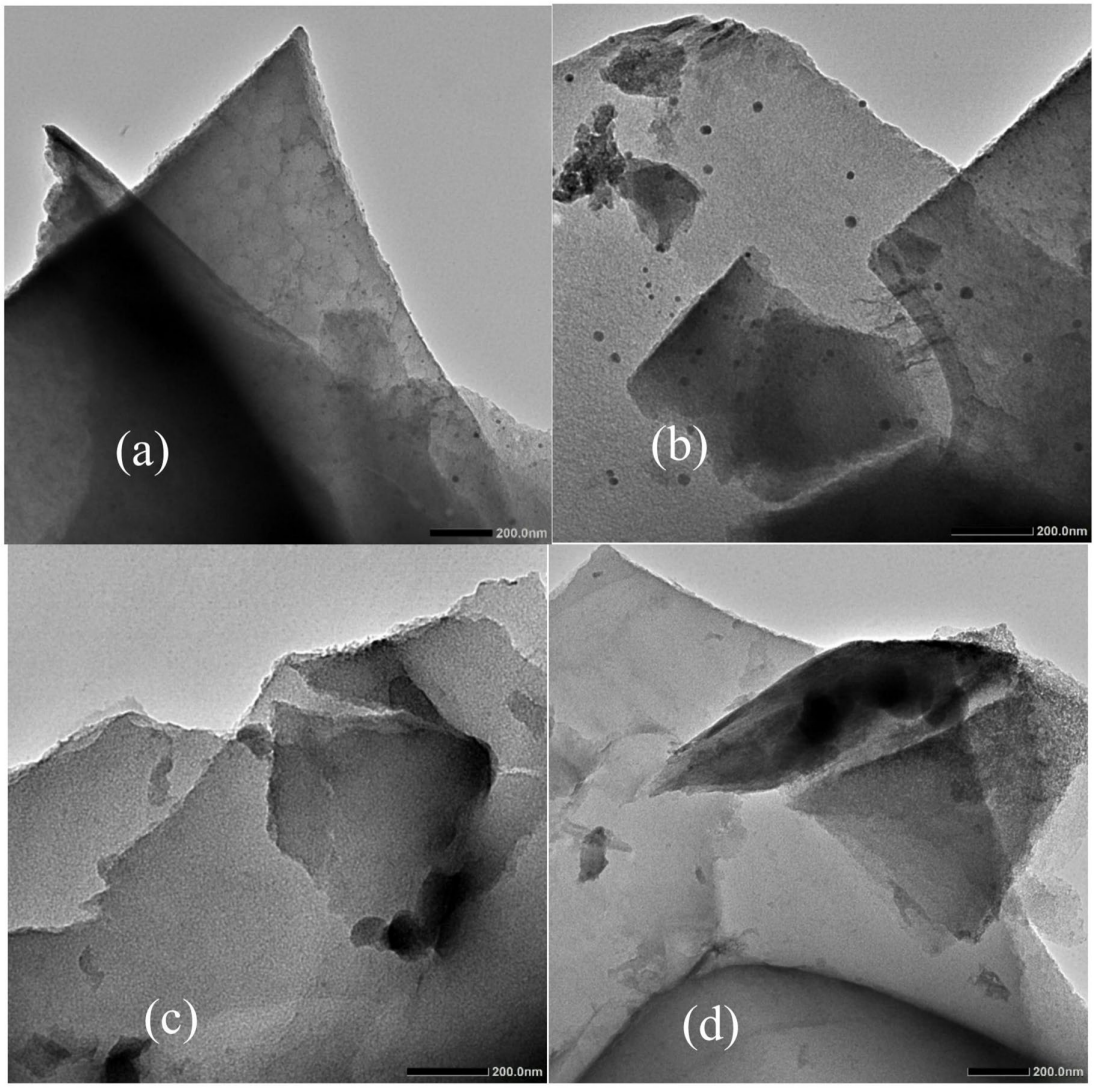
Transmission electron micrographs (TEM) of prepared GNS.

Figure 6a-d illustrates the highly exfoliated, ultrathin morphology of the carbon matrix, highlighting electron-transparent nanosheets characterized by defined angular and triangular profiles. Variations in electron-beam contrast across the sample reveal pronounced surface corrugation and overlapping sheets, which form an interconnected, wrinkled network that inherently prevents layer restacking. Additionally, a fine, mottled contrast pattern distributed across the basal planes indicates a well-developed network of internal micropores and mesopores, while the rough, disordered edge boundaries signify a high density of exposed active defect sites. Dotted across the sheet surfaces and perimeters are fine, nanometer-scaled dark spherical clusters representing dense graphitic domains or trace inclusions inherited from processing. This open, highly accessible, and porous architecture is structurally optimized for supercapacitor configurations, providing unhindered pathways for rapid electrolyte ion migration and maximum double-layer charge accumulation at the interface.

#### Textural properties of electrode material

The active surface area *analysis* and pore structure of the obtained HD-GNS were acquired by N_2_ adsorption/desorption. BET-specific surface area $\:{S}_{BET}$ was found to be 1427 m^2^ g^− 1^ for 1.83 nm pore diameter and 1.91 nm average particle diameter. Moreover, the total pore volume V_t_ calculated from the amount of adsorbed nitrogen at a relative pressure (P/P◦) of 0.99 for the prepared graphene nanosheet reaches 0.654 cm³/g for pores of a diameter smaller than 237.54 nm.

These results are compared successfully with 3D graphene layers with a specific surface area of 532.8 m^2^g^[− 143^, few-layer graphene with 35.5 m^2^g^[− 157^, graphene nanosheets with a specific surface area of 495 m^2^g^[− 145^, rGO with 166 m^2^g^[− 158^, nitrogen-doped graphene with 190 m^2^g^[− 159^, and nitrogen-doped rGO 431 m^2^g^[− 160^. An expanded surface area directly correlates with an increased availability of electroactive sites, which are crucial for the accumulation of charge. This proliferation of active sites is fundamentally important for energy storage applications, as it leads to a direct enhancement in the material’s specific capacitance^1^. On the other hand, these results compared with the activated carbon produced from the same biomass (Honey) were 1664 m^2^g^− 1^ specific surface area, a porous structure of 0.87 cm^3^g^− 1^ a pore volume^41^, 1554 m^2^g^− 1^ with a pore volume of 0.71 cm^3^g^[− 142^. These findings indicated that graphene nanosheets exhibited a reduced pore size and a uniform, random arrangement of aggregates with a closely packed thin layer.

**Fig. 7:**
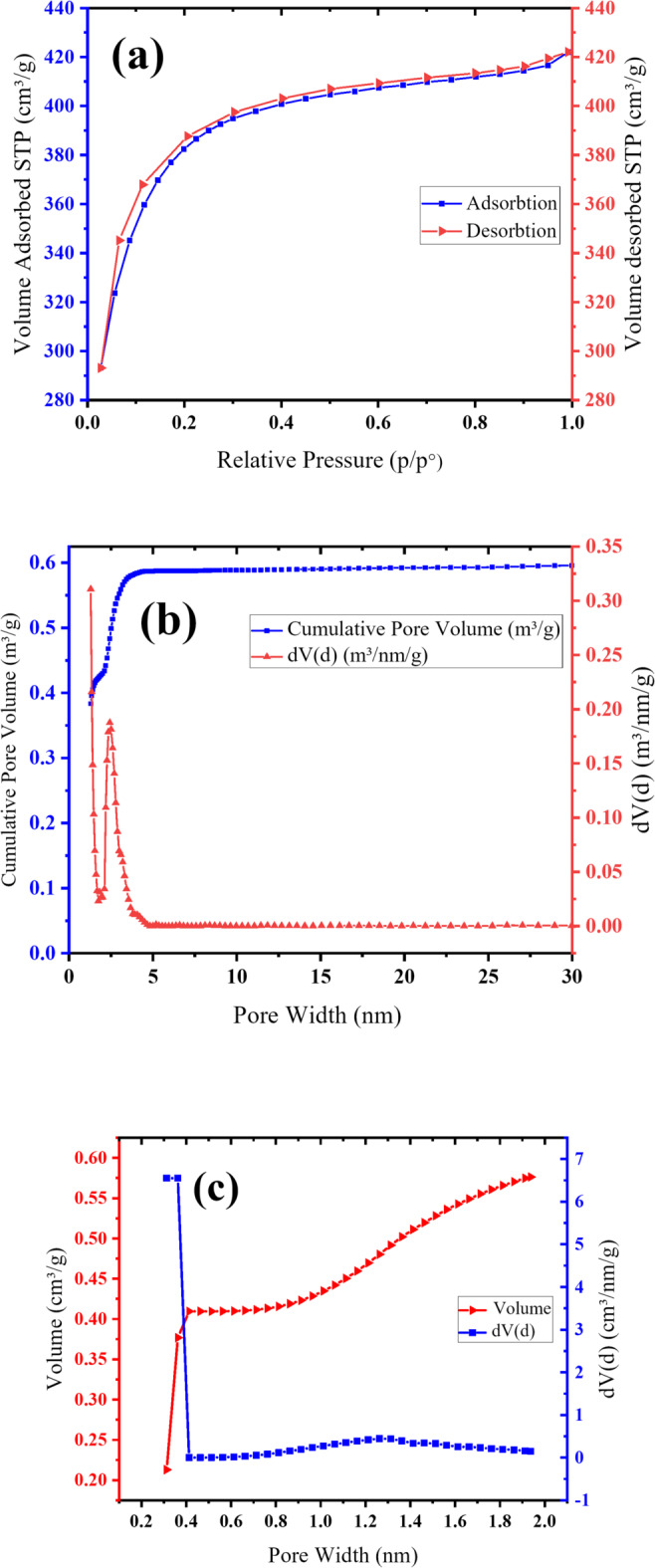
(**a**) Relative pressure with volume adsorption, (**b**) (DFT) model for pore size distribution, and (**c**) is pore size and pore volume distribution (HK) method.

The results are shown in Fig. 7a, the initially steep rise at the relative pressure curve from 0.025 to 0.3 is due to the existence of micropores and exhibits a type (II) isotherm according to IUPAC Taxonomy. All isotherm results, including the total pore volume, BET-specific surface area, micropore volume, and mesopore volume of HD-GNS, are summarized in Table 3. Figure 7b shows the evaluated (DFT) model, density functional theory, is used to depict the pore size distribution of graphene sheets. Also, Fig. 7c describes the pore size and pore volume distribution using the Horvath-Kawazoe (HK) method.

**Table 3:** The total pore volume, BET-specific surface area, and micropore volume using the BET and DFT methods.

$\:{\mathrm{D}}_{\mathrm{B}\mathrm{E}\mathrm{T}}\left(\mathrm{n}\mathrm{m}\right)$	$\:{\mathrm{S}}_{\mathrm{B}\mathrm{E}\mathrm{T}}\left({\mathrm{m}}^{2}{\mathrm{g}}^{-1}\right)$	$\:{\mathrm{V}}_{\mathrm{B}\mathrm{E}\mathrm{T}}\left(\mathrm{c}{\mathrm{m}}^{3}{\mathrm{g}}^{-1}\right)$	$\:{\mathrm{D}}_{\mathrm{D}\mathrm{F}\mathrm{T}}\left(\mathrm{n}\mathrm{m}\right)$	$\:{\mathrm{S}}_{\mathrm{D}\mathrm{F}\mathrm{T}}\left({\mathrm{m}}^{2}{\mathrm{g}}^{-1}\right)$	$\:{\mathrm{V}}_{\mathrm{D}\mathrm{F}\mathrm{T}}\left({\mathrm{c}\mathrm{m}}^{3}{\mathrm{g}}^{-1}\right)$
1.8337	1427	0.6544	1.299	1118.67	0.5975

#### Zeta potential and particle size

**Fig. 8:**
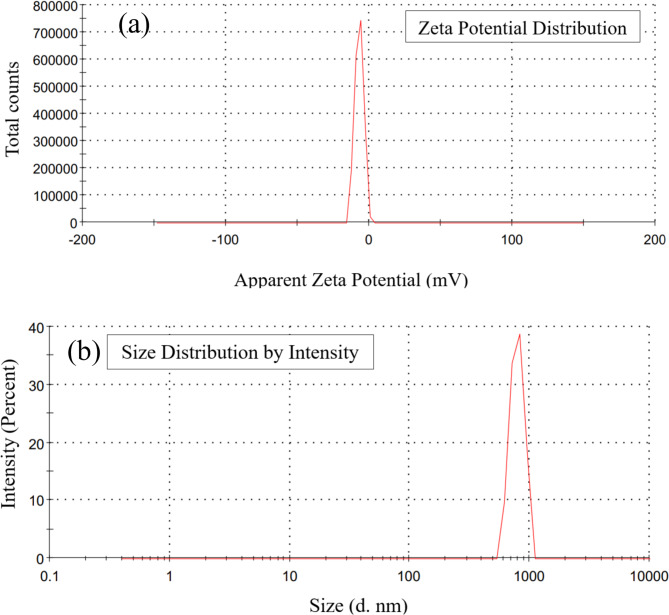
(**a**) zeta potential, (**b**) particle size distribution.

For the HD-GNS samples suspended in an aqueous solution, ZP results (Fig. 8a) ranged from 0 mV to -16.1 mV, with a peak at -6.96 mV when measured at 25 °C. This increased negative charge signifies higher electrostatic repulsive interactions, contributing to enhanced slurry stability and reduced particle agglomeration. This stability is further supported by the material’s very small particle size, which correlates with increased viscosity. Additionally, Fig. 8b indicates the particle size of the layered material grains is 789.7 nm.

### Electrochemical properties

The honey-derived graphene nanosheet electrodes of the graphitic and interwoven structure, together with their enhanced porosity, are predicted to result in outstanding electrochemical capacitive characteristics in a liquid electrolyte, as investigated in 0.5 M $\:{Na}_{2}{SO}_{4}$ solution.

#### Galvanostatic charge-discharge

**Fig. 9:**
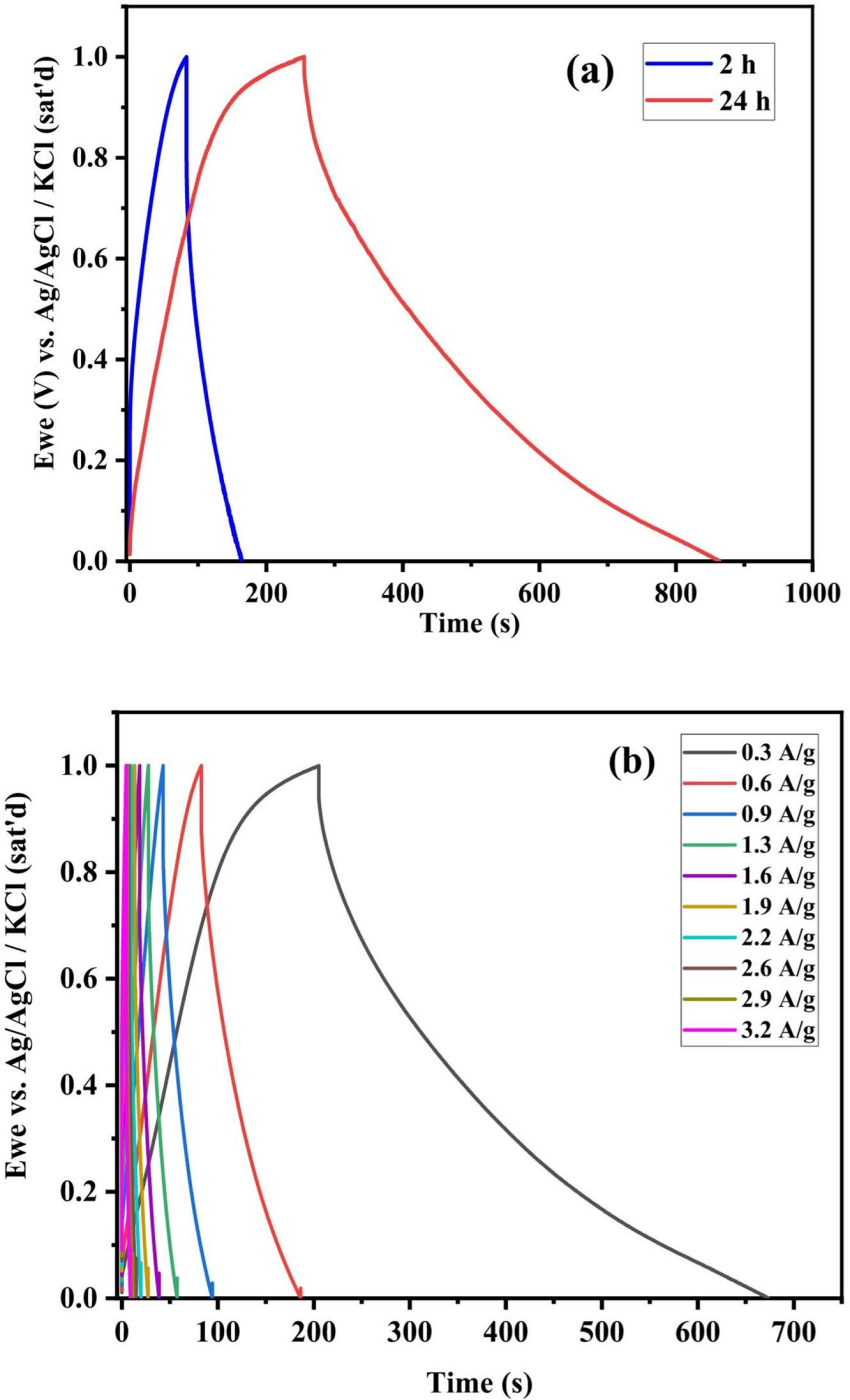
(**a**) GCD curves of electrodes dried for 2 h and 24 h at a current density of 0.3 F/g, (**b**) GCD curves for electrodes dried for 24 h at different current densities.

As depicted in Fig. 9a, HD-GNS electrodes consistently exhibit symmetrical, semi-equilateral triangular GCD curves across various current densities, providing strong evidence of their ideal capacitive behaviour and highly efficient charge storage and release mechanisms. This exceptional electrochemical performance is further highlighted by the highest specific capacitance achieved: 240 F g⁻¹ at an actual current of 1 mA and a current density of 0.3 A g⁻¹.

A particularly significant finding is the minimal IR discharge drop, especially noticeable in the electrode dried for 24 h. This indicates remarkably low internal resistance and improved electrical conductivity, properties that are indispensable for high-power applications. The outstanding performance of the 24-hour dried electrode underscores the critical role of the drying process in significantly enhancing capacitive performance. Appropriate drying directly impacts the accessibility of active sites and optimizes ion diffusion kinetics within the electrode’s porous structure. Consequently, meticulous electrode fabrication, particularly regarding drying parameters, is essential for maximizing the potential of novel carbon materials. Notably, the specific capacitance attained by our honey-derived graphene nanosheets is competitive with, or even surpasses, values reported for other biomass-derived activated carbons and graphene-based materials in aqueous electrolytes, highlighting its promising application in supercapacitors.

Further analysis of the GCD data allowed for the determination of crucial performance metrics: specific energy density (E) and specific power density (P) for the HD-GNS electrodes. Specific energy (E, in Wh kg⁻¹) was computed using the formula

6 $$\:E\:=\:\frac{1}{2}*\frac{1}{3.6}\:C{V}^{2}\:\left(Wh\:{kg}^{-1}\right)\:,$$

while specific power (P, in W kg⁻¹) was derived from

7 $$\:P\:=\frac{E*3600}{\varDelta\:t}\:\:({\rm W}\:{\rm kg}^{-1}),$$

where V represents the voltage window, and Δt denotes the discharge time.

**Fig. 10:**
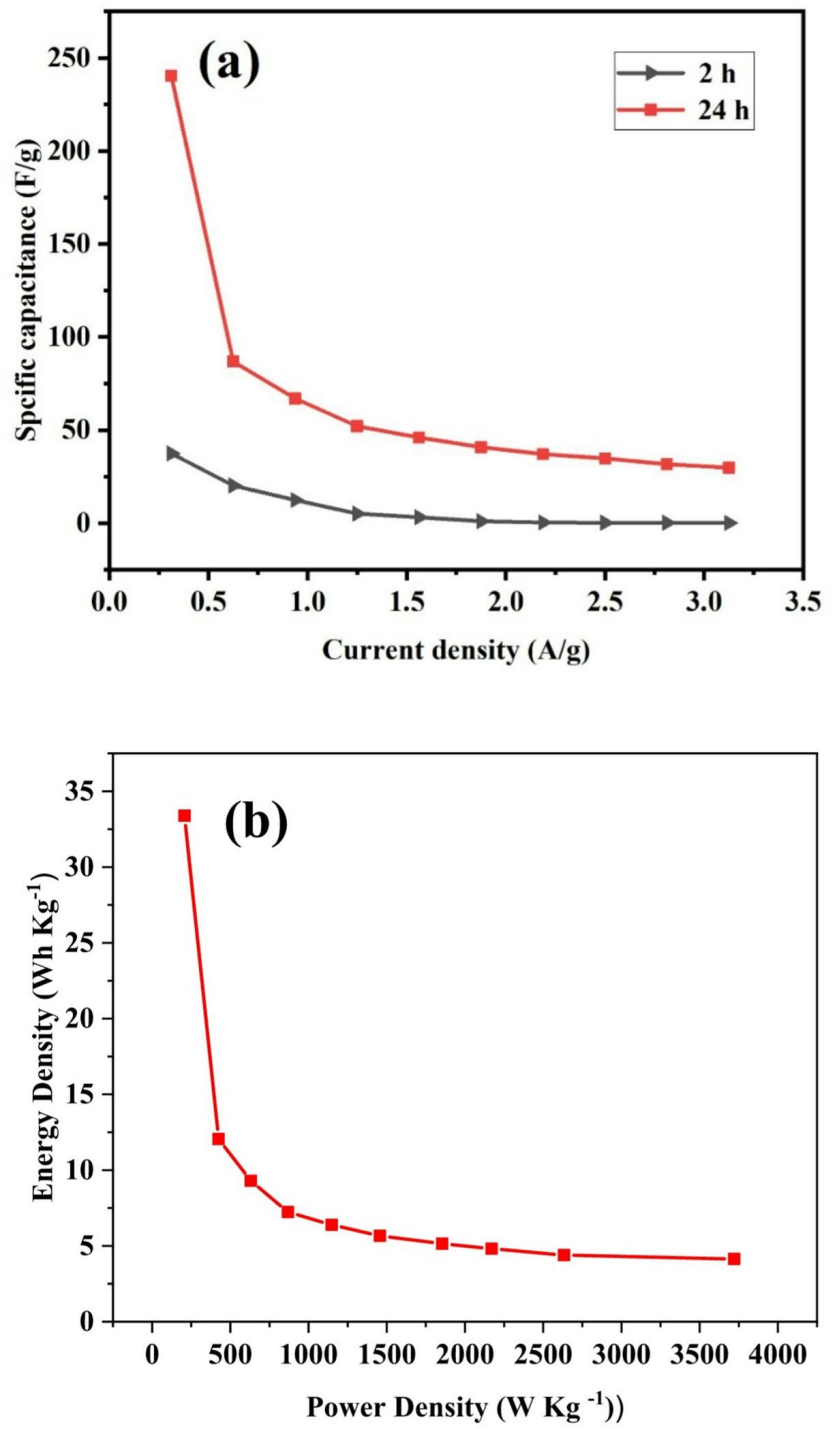
(**a**) Specific capacitance $\:{\mathrm{C}}_{\mathrm{s}\mathrm{p}}$ versus current density for electrodes dried for 2 h and 24 h, (**b**) Ragone plot for electrodes dried for 24 h.

The 24-hour dried electrode demonstrated outstanding performance. Examination of its charge-discharge curves at various current densities (Fig. 9b) revealed a remarkably modest IR discharge drop, signifying impressively low internal resistance. This electrode achieved a maximum specific capacitance of 240 F g⁻¹ at a current density of 0.3 A g⁻¹, as further illustrated in Fig. 10a, which compares specific capacitance versus current density for both 2-hour and 24-hour dried samples. The substantial increase in specific capacitance for the 24-hour dried sample is primarily attributed to the more complete evaporation of residual N-Methyl-2-pyrrolidone (NMP) solvent from the electrode’s porous structure. This thorough solvent removal effectively prevents pore blockage, thereby enhancing the accessibility of the active material’s surface area to electrolyte ions. Additionally, prolonged drying fosters improved structural integrity and better adhesion of the active material to the current collector, both of which are critical for facilitating more efficient charge transport within the electrode assembly.

Operating at a current density of 0.3 A g⁻¹, this 24-hour dried electrode achieved a maximum specific energy density of 33.4 Wh kg⁻¹ at a corresponding specific power density of 207.9 W kg⁻¹. These impressive values unequivocally demonstrate the material’s capability to deliver both high energy and high power. The relationship between energy and power densities is comprehensively visualized in the Ragone plot (Fig. 10b), providing a comparative overview of our HD-GNS performance against other reported supercapacitor materials and showcasing its competitive energy-power characteristics.

For comparison, Zhang et al. reported a specific capacitance of 10.8 F g⁻¹ at 1 A g⁻¹ for pristine reduced graphene oxide (rGO). They significantly enhanced the capacitance to 1093 F g⁻¹ at the same current density by incorporating nickel oxide (NiO) to form an rGO/NiO composite^61^. Table 4 provides a summary of the electrochemical properties for various graphene-based electrode materials. The overall performance of HD-GNS stands out as particularly promising for an electrode material, especially considering it achieves competitive results without requiring additional conductivity or capacitance-enhancing additives. This comparison distinctly underscores the favorable performance of our eco-friendly honey-derived material in supercapacitor applications.

**Table 4:** Electrochemical properties of different graphene-based electrode materials.

Electrode active material	Specific capacitance F g^− 1^	Current density A g^− 1^	Electrolyte	Reference
rGO / acetylene black	10.8	1	6 M KOH	^61^
rGO/NiO / acetylene black	1093	1	6 M KOH	^61^
GNS / carbon black	245	0.05	1 M Na_2_SO_4_	^45^
GNS / carbon black	254	0.05	3 MKOH	^45^
GNS / carbon black	272	0.05	1 M H2SO4	^45^
(NiO/rGO)	171.3	0.5	6 M KOH	^62^
Porus carbon / G Vitamin C	176	20	-	63
Ni-Co / G	233	1	-	64
CoMoS_4_/rGO	68	1	1 M KOH	65
LaFeO_3_/rGO	367.4	1	2 M KOH	^66^
KCdCl_3_/rGO	775	1	1 M KOH	^1^
AC commercial/graphite powder	74.5	0.46	1 M Na_2_SO_4_	^67^
	957.8	0.46	1 M Na_2_SO_4_@ 0.08 M KBr	
HD-GNS	240	0.3	0.5 M Na_2_SO_4_	This work

#### Cyclic voltammetry (CV)

**Fig. 11:**
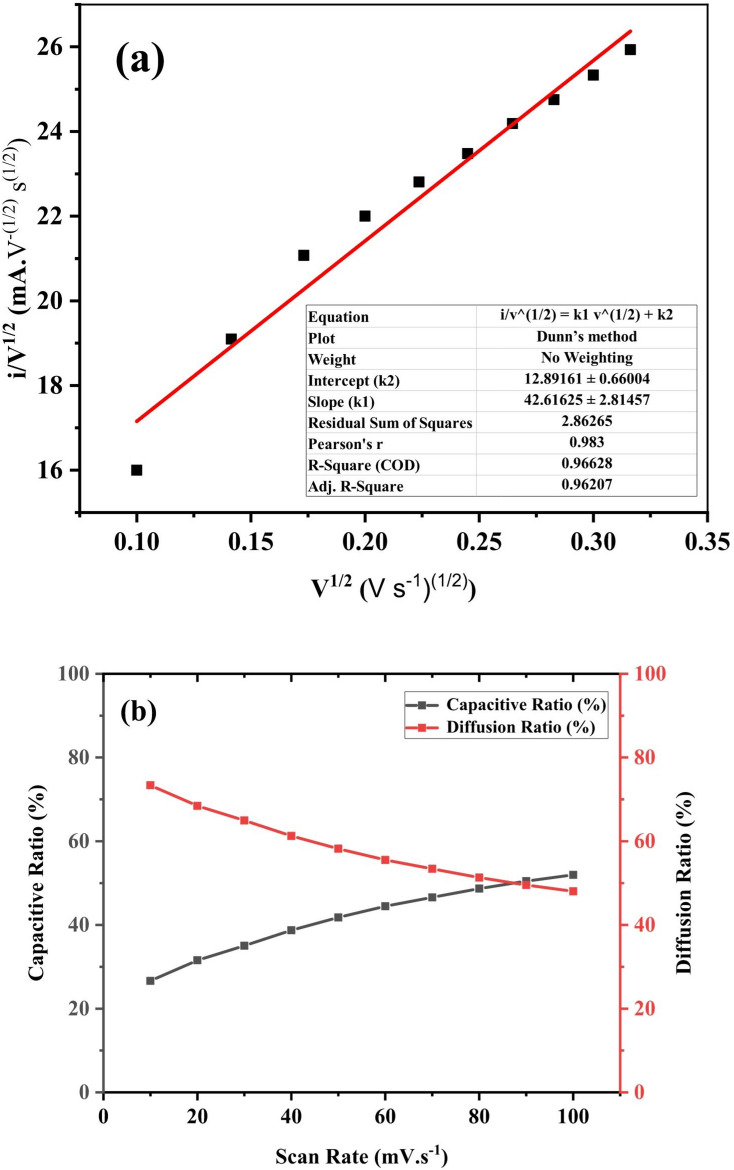
(**a**) Linear fit of $\:\frac{\mathbf{i}\left(\mathbf{V}\right)}{{\mathbf{v}}^{1/2}}$ versus $\:{\mathbf{v}}^{1/2}$ using Dunn’s method for kinetic deconvolution, (**b**) capacitive and diffusion charge storage contribution ratios vs. scan rate.

**Fig. 12:**
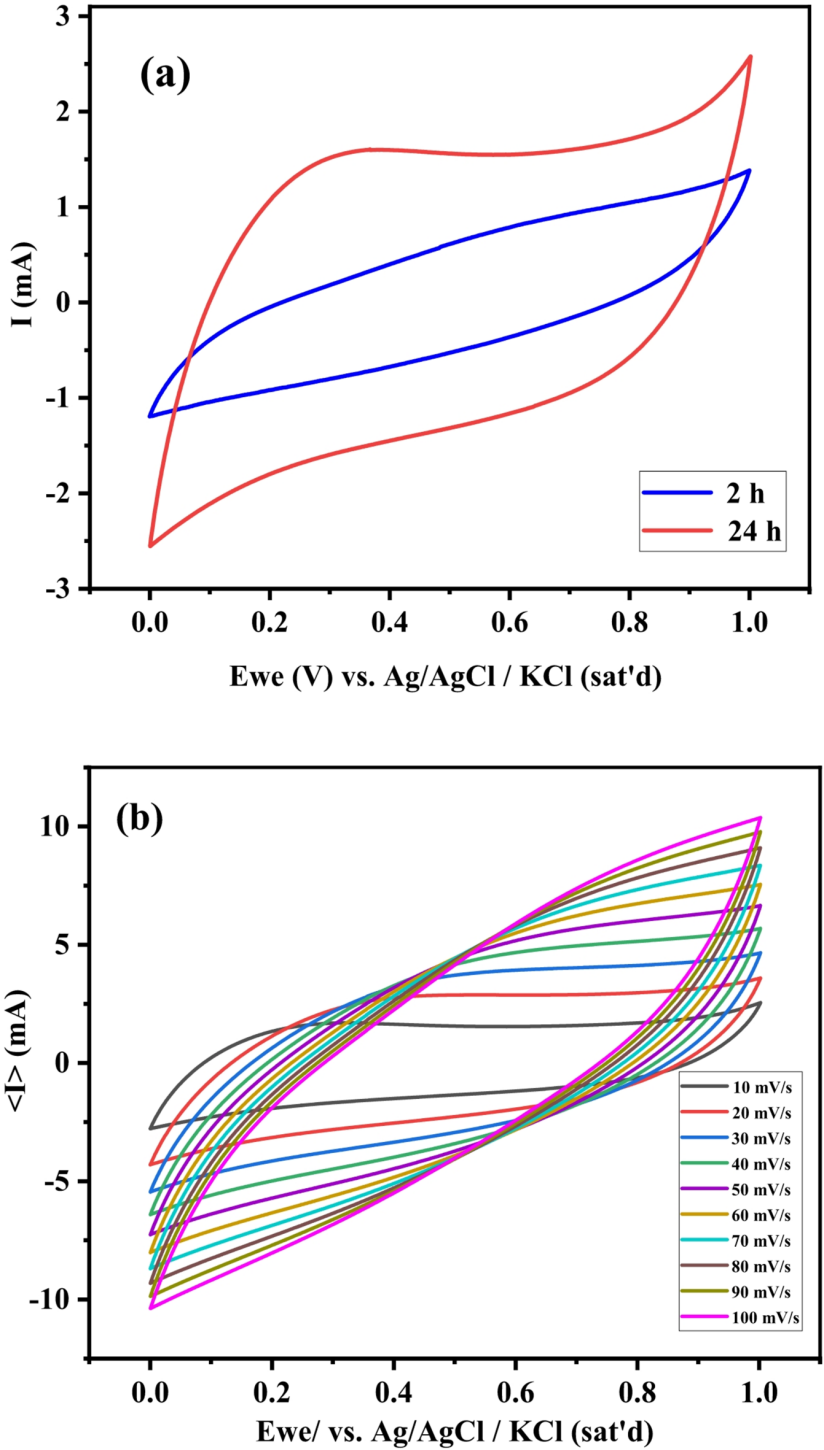
(**a**) CV curves for electrodes dried for 2 h and 24 h at a scan rate of 10 mV/s, (**b**) CV curves for electrodes dried for 24 h at different scan rates.

The prepared HD-GNS electrode represents a hybrid capacitance, demonstrating behaviour consistent with both Electrical Double-Layer Capacitors (EDLCs) and pseudocapacitors. Figure 10a presents a comparison of CV curves for electrodes dried for 2 h and 24 h, both measured at a scan rate of 10 mV sec^− 1^ (Fig. 11). A key observation from Fig. 12a is that the 24-hour dried sample’s CV curve displays noticeable redox humps in both its anodic and cathodic sweeps, a feature significantly less prominent in the 2-hour dried sample. These distinct redox characteristics are indicative of pseudocapacitive contributions, which stem from the reversible redox reactions of the oxygen-containing functional groups located on the graphene nanosheet surface.

As substantiated by the XPS analysis (Fig. 3; Table 1), the HD-GNS material is endowed with a variety of oxygen functionalities (e.g., C-OH, C-O-C, C = O). The more pronounced humps observed in the 24-hour dried sample suggest enhanced accessibility of these electrochemically active sites to the electrolyte ions. This improvement is likely a direct consequence of more complete solvent removal and a more optimized electrode structure, which together facilitate more efficient charge transfer and pseudocapacitive reactions. Conversely, the 2-hour dried sample, potentially retaining residual solvent or exhibiting a less stable structure, may impede access to these active sites, thereby leading to suppressed redox activity.

Additionally, samples dried for 24 h were further investigated across a range of scan rates, as depicted in Fig. 11b. Consistently, the CV curves maintained their nearly rectangular shapes at the specified scan rates, a hallmark of ideal capacitive behaviour.

**Fig. 13:**
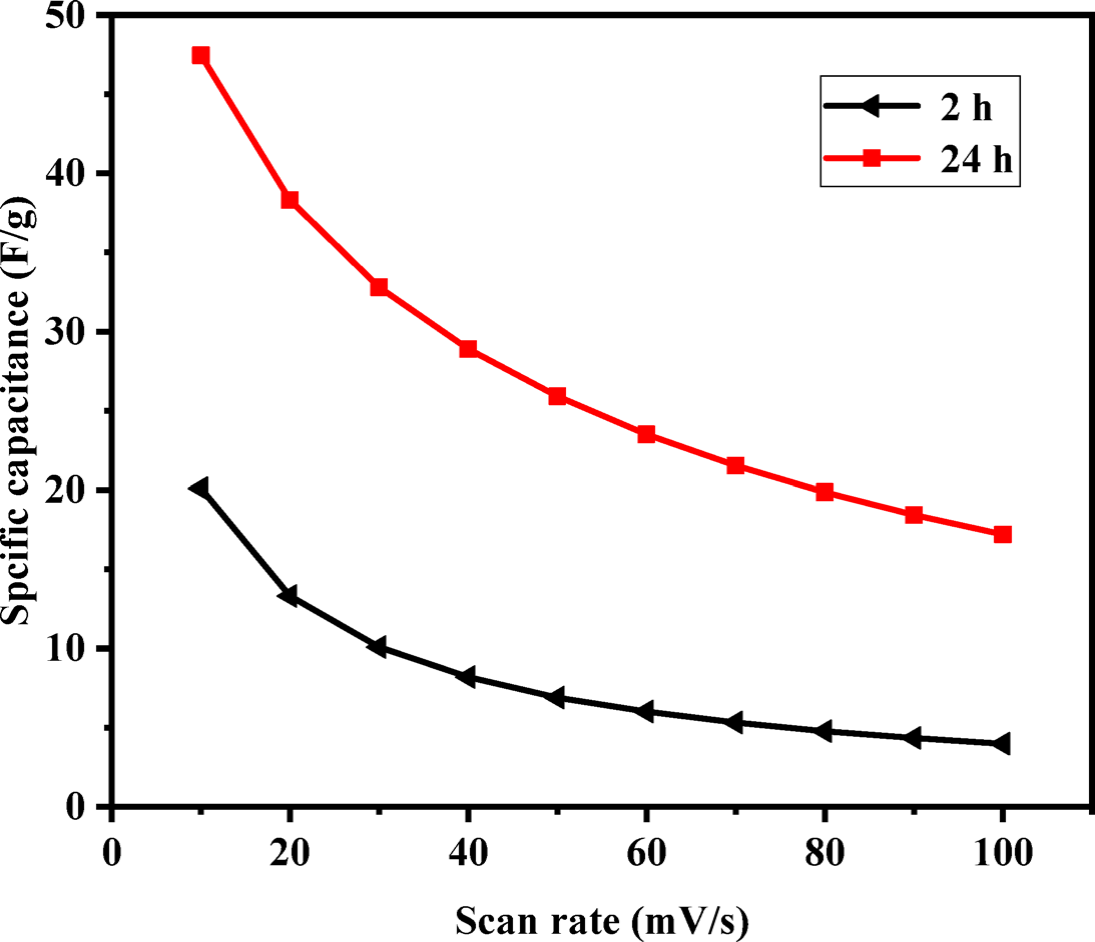
Specific capacitance $\:{\mathrm{C}}_{\mathrm{s}\mathrm{p}\:}$at different scan rates for electrodes dried for 2 h and 24 h.

To further clarify the impact of drying time and scan rate, Fig. 13 summarizes the specific capacitance ($\:{C}_{sp\:}$) at various scan rates for both the 2-hour and 24-hour dried electrodes. As expected, the specific capacitance typically decreases with increasing scan rate. This phenomenon occurs because higher scan rates restrict the ability of electrolyte ions to fully penetrate the deeper regions of the electrode material.

As shown in Fig. 12b, the CV profiles do not distort at high scan rates and remain rectangular. This confirms that the HD-GNS electrodes enable rapid ion diffusion and stable rate performance, making them highly effective as supercapacitor electrodes. While a decrease in specific capacitance with increasing scan rate is commonly observed (Fig. 13), the 24-hour-dried sample exhibits remarkably high capacitance retention at higher scan rates. This suggests the presence of an optimized pore network and highly efficient ion transport pathways within the material.

The combined evidence from both CV and GCD analyses strongly indicates that the straightforward synthesis method, coupled with an optimized electrode drying process, has successfully produced a graphene nanosheet material. This material possesses a highly accessible surface and efficient charge storage mechanisms, positioning it as a promising candidate for high-performance supercapacitor applications.

To profoundly understand the energy storage mechanism and evaluate the exceptional rate performance of the HD-GNS electrode, a detailed kinetic analysis was performed by decoupling the capacitive and diffusion-controlled current responses. The total current ($\:\boldsymbol{i}$) at a given potential ($\:V$) can be expressed as the sum of surface-controlled capacitive processes (encompassing electric double-layer capacitance [EDLC] and fast surface pseudocapacitance) and diffusion-controlled intercalation mechanisms according to Dunn’s relation:

8 $$\:\boldsymbol{i}\left(\boldsymbol{V}\right)=\:{\boldsymbol{k}}_{1}\boldsymbol{v}\:+\:{\boldsymbol{k}}_{2}{\boldsymbol{v}}^{1/2}$$

where K_1_ and K_2_ are potential-dependent constants, and $\:v$ is the scan rate. To resolve these individual constants from the experimental cyclic voltammetry (CV) profiles, the power-law equation was linearized by dividing both sides by $\:{\boldsymbol{v}}^{1/2}$:

9 $$\:\frac{\boldsymbol{i}\left(\boldsymbol{V}\right)}{{\boldsymbol{v}}^{1/2}}={\boldsymbol{k}}_{1}{\boldsymbol{v}}^{1/2}+{\boldsymbol{k}}_{2}$$

By tracking the voltammetric current values across the complete matrix of scan rates (10 to $\:100{\mathrm{\:mV\:s}}^{-1}$) at the steady-state midpoint potential of 0.5 V (vs. Ag/AgCl), a linear relationship of $\:\frac{\boldsymbol{i}}{{\boldsymbol{v}}^{1/2}}$ as a function of $\:{\boldsymbol{v}}^{1/2}$ was constructed, as illustrated in Fig. 11a.

The experimental data points demonstrate an exceptional linear convergence ($\:{R}^{2}\:=0.96628$), indicating that the chosen multi-component kinetic model is highly precise and physically validated for the HD-GNS matrix. From this linear regression analysis, the capacitive proportionality slope K_1_ was resolved to be $\:42.616\pm\:2.815\mathrm{\:mA}\cdot\:{\mathrm{V}}^{-1}\cdot\:\mathrm{s}$, while the diffusion-controlled intercept K_2_ was determined to be $\:12.892\pm\:0.660\mathrm{\:mA}\cdot\:{\mathrm{V}}^{-1/2}\cdot\:{\mathrm{s}}^{1/2}$. The prominent magnitude of the capacitive slope, K1, relative to the diffusion intercept, K2, provides clear quantitative evidence that rapid, surface-confined active pathways heavily govern the electrochemical charge accumulation within the HD-GNS electrode.

The contribution ratio was determined quantitatively across the investigated sweep range. As illustrated in the stacked chart in Fig. 11b, the surface-controlled capacitive mechanism contributes $\:26.63\%\:$of the total charge storage at a low sweep rate of $\:10{\mathrm{\:mV\:s}}^{-1}$. As the scan rate escalates to $\:100{\mathrm{\:mV\:s}}^{-1}$The capacitive contribution increases steadily to $\:51.96\%.$.

This trend is typical for highly conductive carbon nanomaterials; at accelerated scan rates, diffusion-controlled processes become increasingly constrained by mass transport time limits, leaving the highly accessible outer surface area of the exfoliated graphene nanosheets to serve as the primary pathway for rapid ion adsorption/desorption. This high surface-controlled ratio directly corroborates the superior high-rate capability observed for the HD-GNS material.

#### Electrochemical impedance spectroscopy (EIS)

**Fig. 14:**
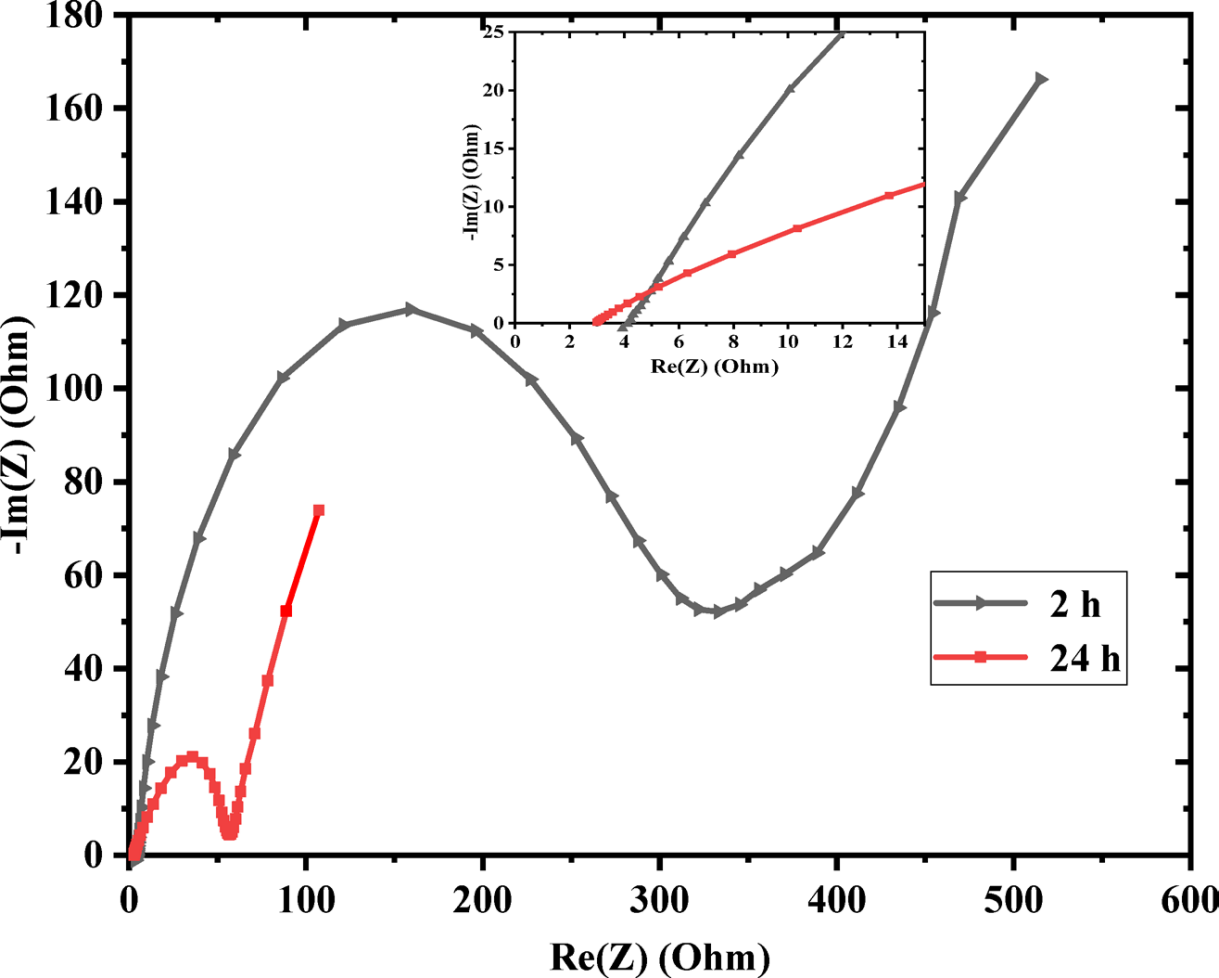
The observed Nyquist plots for electrodes dried for 2 h and 24 h.

EIS analysis was conducted using alternating current frequencies spanning from 100 kHz down to 10 mHz. The resulting Nyquist plots for the electrodes, shown in Fig. 14, reveal a distinct straight line in the low-frequency region. This linear segment indicates the diffusion of ions occurring at the electrolyte/electrode interface. The charge transfer resistance $\:{R}_{ct}$ is expressed by the diameter of the semicircle that the HD-GNS samples exhibit in the high-frequency region. As detailed in Table 4, the electrode dried for 24 h demonstrates a remarkably lower charge transfer resistance ($\:{R}_{ct}$ ), measured at 54.9 Ω, in stark contrast to the 2-hour dried electrode’s $\:{R}_{ct}$ of 335.8 Ω. This significant reduction in $\:{R}_{ct}$ The longer-dried sample further corroborates the advantages of thorough solvent removal. A diminished $\:{R}_{ct}$ signifies more rapid charge transfer kinetics at the electrode-electrolyte interface, which facilitates more efficient ion adsorption and desorption processes during charge-discharge cycles. This property, along with a lower equivalent series resistance (ESR), drives the heightened electrochemical metrics observed in the 24-hour dried electrode configuration. Additionally, the measurement of electrode conductivity is dependent on the equivalent series resistance (ESR) for electrodes, as expressed for each sample in Table 5.

**Table 5:** Values of ($\:{\mathrm{R}}_{\mathrm{c}\mathrm{t}}$) and (ESR) for electrodes dried for 2 h and 24 h.

Time of drying (hrs.)	$\:{R}_{ct}\:\left({\Omega\:}\right)$	ESR $\:\left({\Omega\:}\right)$
24	54.9	3.03
2	335.8	3.25

**Fig. 15:**
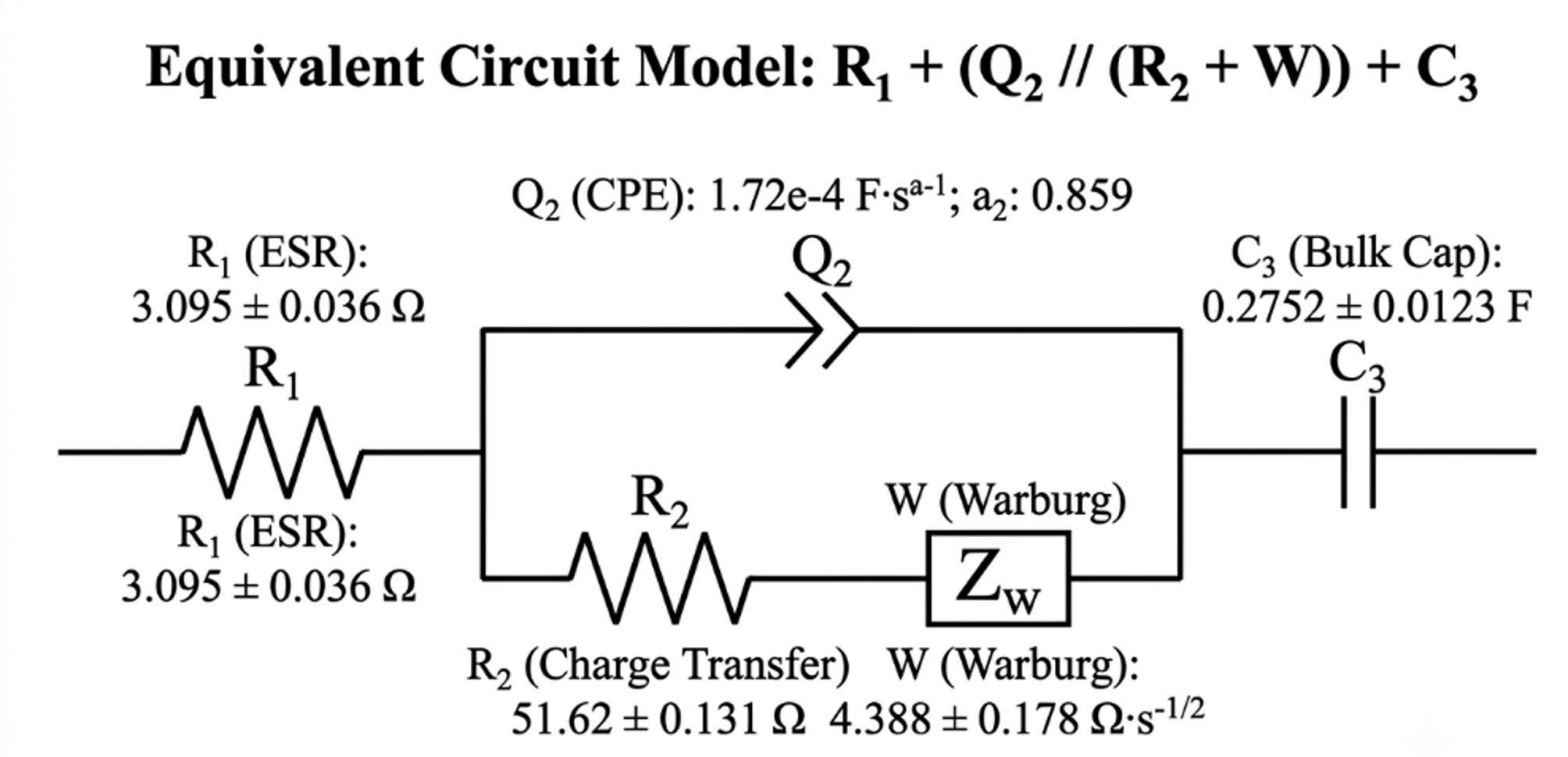
The equivalent circuit for the fitted curve.

To gain deeper insights into the charge transfer kinetics, ion diffusion behavior, and capacitive mechanisms at the electrode-electrolyte interface, electrochemical impedance spectroscopy (EIS) was performed. The resulting experimental Nyquist plot was systematically modeled by complex non-linear least-squares (CNLLS) fitting using the Levenberg-Marquardt approach. The experimental data were successfully simulated using the equivalent circuit configuration designated as R_1_ + Q_2_ / (R_2_ + W_2_) + C_3_, as illustrated in the inset of Fig. 15.

The quantitative values obtained from the fitting, along with their respective standard errors and calculated relative percentage errors, are comprehensively summarized in Table 6.

**Table 6:** Final fitted parameters, standard errors, and physical interpretation of the equivalent circuit elements.

Circuit element	Fitted value	Standard error	Relative error (%)	Physical significance
R_1_ ($\:\varOmega\:$)	3.095	0.0272	1.16%	Ohmic/solution resistance
Q_2_ *(*$\:\mathrm{F}\cdot\:{\mathrm{s}}^{a-1}$*)*	$\:1.72\times\:{10}^{-4}$	$\:2.48\times\:{10}^{-6}$	1.44%	Double-layer constant phase element
a_2_	0.8593	0.0025	0.29%	Surface heterogeneity/porosity exponent
R_2_ *(*$\:\varOmega\:$*)*	51.62	0.1310	0.25%	Interfacial charge-transfer resistance
W_2_ $\:({\Omega\:}\cdot\:{\mathrm{s}}^{-\:\frac{1}{2}}\:)$	4.388	0.1780	4.06%	Warburg ion diffusion impedance
C_3_ (F)	0.2752	0.0123	4.47%	Low-frequency accumulation capacitance

The remarkably low normalized chi-squared value confirms the quality and statistical validity of the chosen electrical model ($\:{\chi\:}^{2}/\left|Z\right|=0.04097$), demonstrating an excellent convergence between the simulated trace and the experimental data points. Furthermore, the high precision of the fit is validated by the fact that all individual circuit parameters exhibit relative errors below 5%, ensuring that the model is mathematically stable and avoids artificial over-parameterization.

The solution resistance ($\:{R}_{1}=3.095\:\varOmega\:$) reflects the high ionic conductivity of the electrolyte and minimal contact resistances. The high-frequency semicircle is governed by a parallel combination of the charge-transfer resistance ($\:{R}_{2}=51.62\:\varOmega\:$) and a constant phase element ($\:{Q}_{2}$). The introduction of $\:{Q}_{2}$ instead of a pure double-layer capacitor ($\:{C}_{dl}$) is physically justified by the porous nature and surface roughness of the electrode material, which causes a dispersion of capacitance. This non-ideal capacitive behavior is confirmed by the attenuation factor ($\:{a}_{2}=0.8593$), which deviates from unity.

At mid-to-low frequencies, the linear diffusion characteristic is effectively accounted for by the Warburg coefficient ($\:{W}_{2}=4.388\:\varOmega\:\,\:{\mathrm{s}}^{-1/2}$), which relates to the transport rate of electroactive ions within the porous electrode matrix. Finally, the abrupt transition to a nearly vertical capacitive line at very low frequencies is successfully captured by the bulk pseudocapacitance element ($\:{C}_{3}=0.2752\:\mathrm{F}$), signaling efficient ion accumulation and highly reversible charge storage capability.

### Electrochemical stability and degradation phenomena

Figure 14a, b illustrate the resulting cyclic stability curve, charting both specific capacitance (F/g) and Coulombic efficiency (%) over these extended cycling periods. Electrode stability is a critical parameter for supercapacitor applications, directly assessed through the life-cycling examinations of the prepared electrode, which featured a 0.9 mg mass loading. The electrode’s durability was tested by performing repeated galvanostatic charge-discharge (GCD) cycles at a current density of 1.1 A/g in a 0.5 M electrolyte for both 1,000 and 10,000 charge-discharge cycles.

**Fig. 16:**
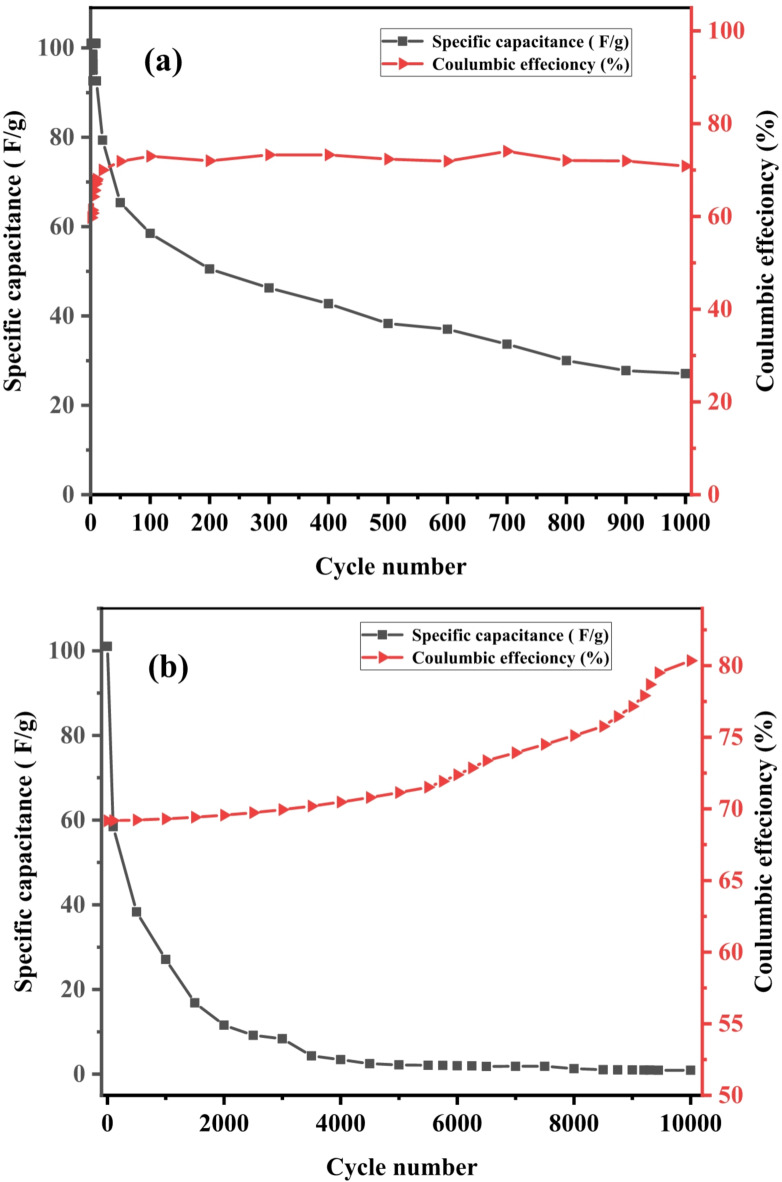
(a, b) The cyclic stability curve versus specific capacitance (F/g) and coulombic efficiency (%) for 1000 and 10,000 GCD Cycles.

The cyclic stability of the prepared electrode, with a mass loading of 0.9 mg cm⁻², was assessed by conducting repeated GCD tests at a current density of 1.1 A g⁻¹ using a 0.5 M Na₂SO₄ electrolyte for both 1,000 and 10,000 charge-discharge cycles. As illustrated in Fig. 16a and b, the specific capacitance of the graphene nanosheet displayed a considerable decline, dropping by 74.1% from its initial capacity after 1000 cycles, ultimately reaching 0.9 F/g. Despite this, the performance also showed a notable improvement in coulombic efficiency, increasing from 69% to 80% after 10,000 cycles. However, this still points to a challenge in maintaining long-term capacitance.

#### Dynamic chemical mechanism of sulfur accumulation (origin and baseline quantification of interfacial contaminants)

The dynamic breakdown of the active material is directly tied to the precursor’s acid-treatment history. Concentrated sulfuric acid (H_2_SO_4_) is utilized during the primary carbonization phase to isolate the carbon framework from the raw honey. Despite subsequent rigorous neutralization and washing with double-distilled water, trace sulphate species (SO_4_^2−^) remain structurally sequestered within the high-surface-area porous matrix (S_BET_ = 1427 m^2^ g^− 1^). This condition is empirically verified by the bulk energy-dispersive X-ray (EDX) spectroscopy data (“Morphological property”, Table 2), which records an initial bulk sulfur level of 0.2 wt% (0.08 at%) embedded inside the pristine carbon matrix before any electrochemical testing.

#### Faradaic reduction mechanism of sulfur passivation

Under continuous cycling, these trapped internal species mobilize inside the dense double layer and undergo sequential Faradaic reduction pathways at the carbon-electrolyte interface. The SO_4_^2−^ ions convert into intermediate sulphites (SO_3_^2−^), ultimately yielding solid-phase elemental sulphur (S_s_, likely in the form of S_8_ rings) via the following equations:

10 $$\:{\mathrm{S}\mathrm{O}_{4}^{2-}\:+\:4\mathrm{H}^{+}\:+\:2\mathrm{e}^{-}\:=\:\mathrm{S}\mathrm{O}_{3}^{2-}\:+\:\mathrm{H}_{2}\mathrm{O}}^{}$$

11 $$\:\mathrm{S}\mathrm{O}_{3}^{2-}\:+\:6\mathrm{H}\hspace{0.17em}^{+}\hspace{0.17em}+\hspace{0.17em}4\mathrm{e}^{-}\:=\:\mathrm{S}\mathrm{s}\hspace{0.17em}+\hspace{0.17em}3\mathrm{H}_{2}\mathrm{O}$$

The precipitation of these insulating sulphur clusters inside the fine micropores and mesoporous channels deactivates the electrode via two distinct modes:

1. It physically clogs the pore network, preventing electrolyte ions from accessing the active internal surface area.
2. It interrupts electronic percolation across the conductive, sp^2^-hybridized graphitic networks (which exhibit a dominant 70.61% C1s XPS contribution), drastically escalating charge-transfer resistance and leading to device failure.

#### Targeted mitigation strategies for enhanced durability

The results reveal the deterioration of the electrode material due to the formation of a yellow sulfur layer on the tested electrode following the successive charging and discharging cycles. This sulfur layer, likely originating from residual sulfuric acid used during the initial carbonization process (even after thorough washing), leads to blockage of active pores, thereby hindering efficient ion transport and charge storage. While some recent studies on graphene-based supercapacitor materials report higher capacitance retention, our identified degradation mechanism highlights a specific challenge inherent to our material’s precursor processing. To enhance the overall performance and stability for future applications, several strategies can be considered. The graphene nanosheets could be combined with other materials, such as highly stable metal oxides (e.g., MnO₂ or TiO₂), or integrated with conductive additive substances (e.g., acetylene black or carbon black). These compounds often provide crucial additional ionic transport routes, improve structural integrity, and enhance overall electrochemical stability. Furthermore, incorporating a redox additive (e.g., Potassium Bromide) into the electrolyte has demonstrated a significant role in improving the electrochemical properties and stability of supercapacitor electrodes, presenting a promising avenue for our honey-derived material^67^.

## Conclusion

Graphene nanosheets were successfully created through the chemical carbonization of natural honey, followed by thermal treatment and activation with potassium hydroxide. The resulting fragile carbon structure underwent continuous grinding and purification before being used to prepare electrodes and for characterization. This research provides unique insights into the structural and electrochemical properties of graphene nanosheets derived from an innovative, eco-friendly biological source, emphasizing honey’s potential as a sustainable, cost-effective precursor for high-performance supercapacitor electrodes.

A key finding of this study is that optimizing a seemingly simple fabrication step—the electrode drying time—is critical for fully realizing these materials’ capacitive potential. While there’s room to improve cyclic stability, pinpointing the degradation mechanism (sulfur layer formation) offers a clear path for future material and interface enhancements, such as integrating metal oxides or conductive additives. Ultimately, this work not only contributes to the development of sustainable energy storage solutions but also provides valuable guidance for the intelligent design and fabrication of next-generation supercapacitor electrodes from renewable resources.

The resulting fine material boasts a high specific surface area (1427 m² g⁻¹) and a substantial pore volume (0.654 cm³ g⁻¹). The electrodes were fabricated using a simple, traditional method, notably without the addition of any conductive enhancers, with a mass loading of 3.2 mg cm⁻². These electrodes were then electrochemically examined at different drying times (2 and 24 h). The best performance was achieved after 24 h of drying, yielding a maximum specific capacitance of 240 F g⁻¹ at a current density of 0.3 A g⁻¹ in an aqueous 0.5 M Na₂SO₄ electrolyte. This optimized material also demonstrated high capability to handle the charge and discharge rate at increasing current densities, making it well-suited for supercapacitor systems.

## Data Availability

The authors declare that the data supporting the findings of this study are available within the paper.
